# Developing and validating a questionnaire to assess an individual’s perceived risk of four major non-communicable diseases in Myanmar

**DOI:** 10.1371/journal.pone.0234281

**Published:** 2021-04-27

**Authors:** Kyaw Swa Mya, Ko Ko Zaw, Khay Mar Mya

**Affiliations:** 1 Department of Biostatistics and Medical Demography, University of Public Health, Yangon, Myanmar; 2 University of Community Health, Magway, Myanmar; 3 University of Public Health, Yangon, Myanmar; Universiti Sains Malaysia, MALAYSIA

## Abstract

Adopting healthy lifestyles is greatly influenced by an individual’s perceived risk of developing non-communicable diseases (NCDs). This study aimed to develop and validate a questionnaire that can assess an individual’s perceived risk of developing four major NCDs. We used the exploratory sequential mixed methods design. The qualitative part developed a questionnaire by two rounds of Delphi expert panels. The quantitative part validated the questionnaire using both exploratory (EFA) and confirmatory factor analysis (CFA). We used separate samples for EFA (n = 150) and CFA (n = 210). The participants were aged between 25–60 years of both sexes with no known history of NCDs, and face-to-face interviews were conducted. First, we generated an 86-item questionnaire based on the health belief model. Two expert panels ensured the questionnaire’s content validity. The experts removed the overlapped items and items that did not represent the specific construct and developed a 51-item questionnaire. Next, we validated the questionnaire. We conducted a parallel analysis to determine the number of factors to be extracted. EFA constituted a five-factor model with 22 high loading items, which extracted 54% of the variance. We run four CFA models (single factor, five-factor, bifactor, and hierarchical) and tested the hypothesized five-factor model. It was found that the 21-item questionnaire (removed one efficacy item due to low loading) was satisfied with good psychometric properties and fitted with observed data in the bifactor model (RMSEA = 0.051, CFI = 0.954, TLI = 0.938, SRMR = 0.054). Hence, an individual’s perceived risk of getting NCDs was constituted with a general perceived risk construct and five specific constructs (perceived susceptibility, perceived barrier, perceived benefit, perceived self-efficacy, and perceived behavioral change intention). It can be measured using the developed questionnaire (NCD-PR5-21). Further research is warranted to assess the questionnaire’s utility in a mismatch between risk perception and current risk; and individualized counseling for behavioral change communication.

## Introduction

The World Health Organization (WHO) estimates that non-communicable diseases (NCDs) are responsible for the death of 41 million people each year and accounts for 71% of all deaths globally [1]. NCDs have become a significant public health problem for developing countries, and it has been recognized as a major challenge to achieving sustainable development goals. The WHO estimated that 8.5 million lives were lost due to NCDs in the South East Asia Region [2]. As one of 23 high burden countries concerning NCDs [3], Myanmar encountered a significant burden and a high potential to increase exposure to major NCDs’ risk factors in the future [4].

According to the WHO 2^nd^ Global Status report, NCDs accounted for more than 50% of Myanmar’s total deaths. The probability of dying from one of the four major NCDs (Cardiovascular diseases (CVDs), Diabetes, Cancer, and Chronic respiratory diseases) was about 24% in individuals between 30 and 70 years of age [5]. The report also highlighted a growing concern for NCDs’ several risk factors, including hypertension and overweight/obesity. The Myanmar STEP survey conducted in 2014 revealed that 12% of the people aged 40–64 years had already had one kind of CVDs or a high level (i.e., ≥30%) of 10-year CVDs risk [6]. These findings support the national concern of growing several risk factors for NCDs.

NCDs are diseases related to an individual’s behavior. Most of the risk factors are modifiable. The perceived risk of developing a particular illness can influence an individual’s motive to take necessary preventive measures against this illness [7]. Hence, an individual’s perception of developing a disease or the prospect of suffering its morbidity is critical in adopting a healthy lifestyle regardless of the disease’s actual risk [8]. An accurate understanding of the risk of getting a disease leads high-risk persons to adopt healthy lifestyles and follow the required preventive interventions [9–11]. It can also reduce low- or average-risk persons’ anxiety not to follow high-cost, sophisticated investigations [12].

Hence, exploring the individual’s perception of developing NCDs using a standardized and validated tool specific to the country context might help address NCDs’ risk factors in Myanmar. The use of questionnaires in social science research has been widely used as a feasible and inexpensive method to measure an individual’s risk perception of specific diseases. Moreover, this method gives more significant power than other methods by including many respondents and enabling statistical analysis [13]. However, a standardized and validated questionnaire that can measure an individual’s perceived risk of developing NCDs does not currently exist. Hence, we aimed to develop and validate a questionnaire that can assess individuals’ perceived risk of developing four major NCDs within Myanmar’s specific socio-economic-culture context.

## Materials and methods

We used an exploratory sequential mixed methods design and conducted this study in 6 phases– 3 phases of questionnaire development and 3 phases of questionnaire validation.

### Questionnaire development

Although the pathogenesis of four major NCDs is different from each other, WHO stated that four major risk behaviors had driven the current rise of NCDs burden in developing countries. These are tobacco consumption, including smoking and betel chewing, the harmful use of alcohol drinking, lack of physical activity, and unhealthy diets [14]. We focused on four major NCDs since these diseases were highly prevalent in Myanmar and related to these common risk behaviors. Hence we included these risk behaviors appropriately in the questionnaire development.

#### Phase 1. Conceptualization of constructs

We conceptualized the NCDs’ perceived risk based on the Health Belief Model (HBM), a famous and widely used health behavioral model in communication research [15, 16]. In brief, the HBM mentioned that health behavior is determined by an individual’s risk perception of the disease. The risk perception is based on the susceptibility and severity of that disease. It is also related to the perception of barriers and benefits of doing available preventive actions, the confidence level to carry out the recommended actions (efficacy), and the willingness to change the recommended behavior (behavioral change intention).

#### Phase 2. Item generation (draft questionnaire) and modification of questionnaire by expert panels (Delphi method) to obtain satisfactory content validity

A thorough literature review was done on PubMed, Google scholar, to identify the items that can assess NCDs’ perceptions, and we found many perceived risk assessment studies. Some of the studies were based on HBM but not for NCDs, and some studies assess the perceived risk of a specific NCD but not based on HBM. Hence, we could not use a published and validated questionnaire for our study; instead, we adapted some question items from two studies [13, 17] that developed and validated the questionnaire for risk perception of NCDs. Then, we generated an 86-item question pool based on the HBM’s constructs (S1 Table). Among the 86-item questionnaire, 25 items assessed the perceived susceptibility construct, nine items for the perceived severity, nine items for the perceived benefit, 12 items for the perceived barrier, 11 items for the self-efficacy, and 20 items for the behavioral change intention. We ensured that the items in the questionnaire reflected aspects of Myanmar’s culture.

The question items were concerned with the background risks, lifestyles, emotions, beliefs and traditions, preventive behaviors such as exercising and medical check-ups, and NCDs’ socio-economic impacts. The susceptibility items were constructed based on the individual’s characteristics such as age, past exposure to risk behaviors (smoking, alcohol drinking). The susceptibility items also included the likelihood of getting NCDs in the near future and cultural-specific fatalism (individual’s belief that there is nothing to do to control the consequences of an outcome) to assess how vulnerable they are to NCDs. The severity items were based on the consequences of NCDs, such as physical, psycho-social, and economic impacts of the diseases. The benefit items were based on recommended healthy lifestyles (doing exercises, eating healthy foods, regular health check-ups) and abandoning the risk behaviors (smoking, harmful alcohol drinking, sedentary lifestyle). The barrier items were concerned with the difficulties encountered doing recommended behaviors such as time constraints, money, social barriers, and lack of knowledge. The self-efficacy items were intended to measure how confident they are to follow the recommended healthy lifestyles and abandon the risk behaviors. The behavioral change intention items were constructed to measure their willingness to change recommended healthy behaviors. We used a four-point Likert scale (strongly disagree, disagree, agree, and strongly agree) for the susceptibility, severity, benefit, barrier, and intention constructs. However, we used a different four-point Likert scale (not at all confident, somewhat confident, moderately confident, and completely confident) for the self-efficacy construct. The questionnaire also included a demographic section that collected the respondents’ age, gender, education, and occupation (See details in S1 Table).

We next assessed the questionnaire’s content validity by using the Delphi method that explored a range of ideas and opinions or reached a consensus on a particular topic. Several studies proved the validity and usefulness of this technique in questionnaire development researches [18–20]. The study invited ten experts: A clinician, Public Health specialists, Epidemiologists, Health policy specialist, Social scientist, Demographer, Public health administrator, and a researcher specialized in NCDs. Eligibility criteria for experts were having at least either a master’s degree or Ph.D. and ten years of services for their professionals. Moreover, the expert panel, including experts from different specialties, can contribute to the study more accurate in detecting the questionnaire’s content validity.

Two rounds of Delphi iterative expert panels were conducted with the experts to achieve consensus regarding the included question items. During the first round of Delphi, the generated 86-item question pool was sent to the experts by email, and the study took consensus on items that should be included in questionnaires. The experts were asked to rate each of the items using three criteria—not at all representative, somewhat representative, or clearly representative, to assess the perceived risk of major NCDs. We removed the items which failed to receive the “clearly representative” response by less than 60% of the experts. The researchers presented the first-round results to the same experts during face to face second Delphi round. The experts also pointed out that some items had a similar meaning; hence, the researcher asked again consensus among experts which items should be included in the questionnaire. Then, experts reviewed the selected items again to ensure the correctness of domain membership and paid particular attention to the items’ wording and sequencing.

#### Phase 3. Pre-testing to modify questionnaires to obtain satisfactory face validity

To obtain face validity, we conducted a pre-test to ensure the questionnaire’s comprehensibility and readability among 15 individuals aged between 25–60 years of both sexes. The selected individuals had no known NCDs and were fluent in Burmese (Myanmar language). Moreover, we asked the participants to comment on the questionnaire items’ clarity, content, appropriateness, and format. Before the final data collection, the questionnaire was edited according to the participants’ suggestions to ensure translational validity (face validity).

### Questionnaire validation

#### Phase 4. Data collection and items purification by exploratory factor analysis

We used consecutive sampling to collect data from 373 participants from the outpatient departments of Yangon General Hospital, North Okkalapa General Hospital, and other teaching hospitals from September to December 2019. They were patient’s attendants, workers, and office staff who met with inclusion criteria. After getting informed consent, we collected data using a pre-tested questionnaire in a room where privacy was ensured. After data collection, we provided the pamphlets (Standardized Health Messages book Page no. 139, 140, 141) regarding NCDs prevention to all participants [21]. We discarded data from 13 participants due to incomplete information. We randomly divided the participants into two groups– 150 participants for exploratory factor analysis (EFA) and 210 participants for confirmatory factor analysis (CFA).

The objective of this phase was to reduce the number and evaluate the robustness of the intended items in the questionnaire. We examined the facility index to assess whether respondents answered the items in the same direction. We recoded the items with reverse scoring to achieve the conceptual direction of the construct. Before EFA, Kaiser-Meyer-Olkin (KMO) measure of sampling adequacy and Bartlett’s test of sphericity (Observed correlation matrix was an identical matrix) assumptions were tested [22].

We conducted an EFA, a widely used technique in exploring theoretical constructs, to determine the questionnaire’s factorial structure and explore which items were collectively constituted a particular construct [23]. Since the study used an ordinal measure of a four-point Likert scale response in the questionnaire, we assessed the polychoric correlation matrix among question items using the FACTOR program (10.10.0) [24]. We also conducted a parallel analysis (the method compares the Eigenvalue generated from the data matrix to the eigenvalues produced from a Monte-Carlo simulated matrix created from random data of the same size) to determine the optimum number of factors to be extracted [25]. We used a parallel analysis scree plot to visualize the number of factors needed to obtain.

The EFA was achieved by using the maximum likelihood factor extraction method with the Promax rotation. The EFA identified the underlying relationship between measured items to constitute the constructs. We also assessed inter-factors correlation. If some factors correlated strongly with each other, i.e., >0.7, the EFA was rerun only with these correlated factors to identify the items correlated with both factors. We removed the items related to both factors to increase discriminant validity. We extracted the factors based on not only factor loading but also the interpretability of the factors. We removed the items with low factor loading <0.40 and cross-loading with the difference below 0.2 at each step of iteration [26]. We also removed items that negatively affected the reliability of latent factors to increase constructs’ reliabilities. The EFA was conducted again, excluding every item deleted for reliability reasons.

#### Phase 5. Confirmatory factor analysis to check constructs validity

To statistically test the EFA proposed hypothesis of perceived risk constructs on developing the NCDs, we ran a CFA with robust unweighted least square estimation method to account ordinal nature of the data [27] from 210 participants. We also assessed the constructs’ convergent and discriminant validity [28] and model fit measures using the Structural Equation Modeling (SEM) technique. We used the root mean square error of approximation (RMSEA <0.08) with 90% CI, RMSEA PCLOSE (>0.05), comparative fit index (CFI >0.92), Tucker Lewis index (TLI>0.92), relative chi-square (χ^2^/df <3), and standardized root mean square residual (SRMR ≤0.08) as model fit indicators [28]. Convergent validity and discriminant validity were assessed by average variance extracted (AVE) by each latent construct and square correlation (SC) among latent variables. Convergent validity is satisfied if the AVE value is greater than or equal to 0.5. The discriminant validity is satisfied if SC values of one construct with other constructs are less than the AVE value of this specific construct [29, 30]. We also ran three alternative CFA models–(i) single-factor, (ii) bifactor, and (iii) hierarchical models and examined the best fit model based on model fit indices for competing models.

#### Phase 6. Assessment of unidimensionality, reliability, and construct replicability of the questionnaire

The questionnaire’s dimensionality was assessed by common variance attributed by the general factor in the bifactor model using the explained common variance (ECV). If the ECV value of the general factor is greater than 0.9, it indicates unidimensionality. If it is lower than <0.7, it indicates multidimensionality of the questionnaire [31]. We also assessed each latent factor’s reliability using omega (Ω) and omega hierarchical (Ωh) indices. These indices are useful to measure the questionnaire’s precision in assessing the general and specific latent constructs, and the value ≥ 0.7 indicates good reliability. We calculated the construct replicability index (H index) of each construct to indicate how the indicator variables represent the latent construct. A high H index (>0.7) indicates the constructs’ stability and less likely to vary across the different samples. Conversely, a low H index indicates the latent constructs were not well-defined by indicator variables and higher chances of variation across studies [32]. We used R software version (4.0.3) and “lavaan” 0.6–7 package in R studio for all analyses [33].

#### Ethical consideration

We conducted the study after getting the approval of the Institutional Review Board of the University of Public Health, Yangon. The Certificate of Approval number was UPH-IRB (2019/Research/7). Voluntary participation was ensured, and written informed consent was taken. The coding system maintained the participants’ confidentiality, and nobody, except the researcher, was allowed access to the data.

## Results

**Fig 1:**
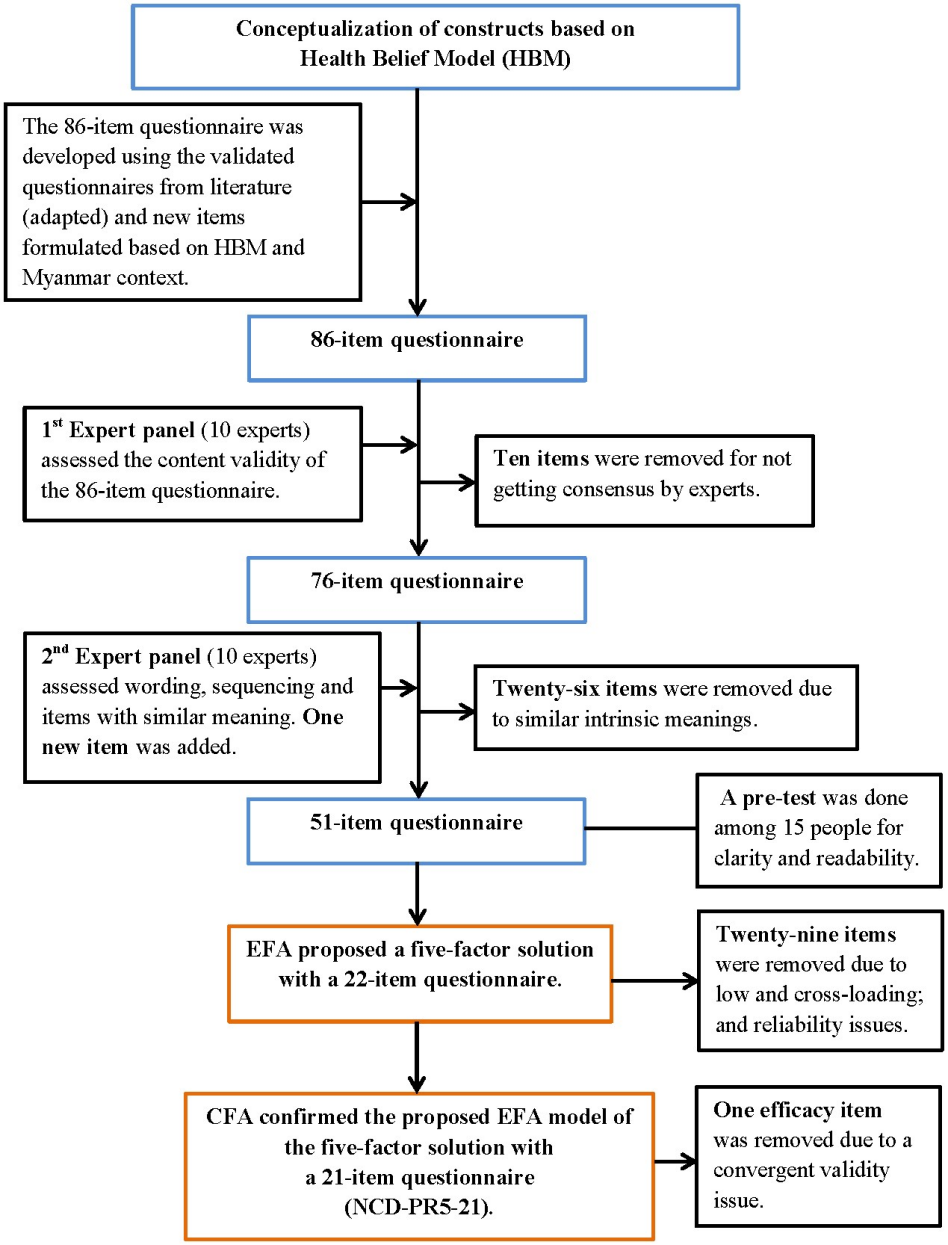
Questionnaire development and validation process flow diagram.

We described the sequential development of the final 21-item questionnaire from an 86-item question pool (Questionnaire development and validation phases) in Fig 1. In brief, we first conceptualized the basic constructs of NCDs’ perceived risk based on the HBM and then developed an 86-item question pool. Two rounds of expert panel satisfied the content validity of the 51-item questionnaire to collect the data from participants. A separate analysis of EFA and CFA proved that a 21-item questionnaire (NCD-PR5-21) was valid and reliable to assess the perceived risk of NCDs among the Myanmar population.

The background characteristics of the respondents in both EFA and CFA were described in the S2 Table. The majority of the participants were below 40 years (66%) and females (74%). About 70% of the participants were university graduates, and 60% were government staff.

### Questionnaire development

We convened two rounds of Delphi expert panels, each consisting of ten members. Our first expert panel assessed the 86-item questionnaire for content validity using pre-defined three criteria, i.e., not at all representative, somewhat representative, or clearly representative. Among 86 items, we removed ten items (Item numbers 8, 9, 11, 13, 16, 20, 29, 32, 48, and 84) that were not accepted as being “clearly represented” by more than 60% of the experts (S1 Table).

The second expert panel noticed that some items had similar intrinsic meaning; therefore, this panel achieved consensus to choose items that accurately reflected the perceived risk of developing major NCDs for specific constructs. Among items of perceived susceptibility construct, the items in five item-groups (1, 4, 6, 7), (2, 21, 22, 23, 24, 25), (3, 17), (10, 14, 15, 16) and (18, 19) had similar meanings in assessing the perceptions. Hence, the experts agreed to select the items (4, 6), (22, 23, 24), (3), (10), and (18). Regarding perceived severity items, items (26, 33) had similar meanings; therefore, the panel selected item 26 to include in the final questionnaire. The experts agreed to select perceived benefit items 35 and 39 from the similar item groups (35, 38) and (36, 39). There was no similar issue in the perceived barrier domain. Among self-efficacy items, the item numbers (56, 60, 61) were similar. After getting a consensus, the experts agreed to retain items 60 and 61. They removed item 58 since this item intends only to ask NCDs’ overall preventive measures since many items assess specific preventive measures in the questionnaire. Regarding behavioral change intention items, item number groups (67, 73, 76, 80), (68, 70), (69, 71, 79), (72, 75, 77, 81), (74, 78), (82, 83) had similar meaning, hence, only items 67, 68, 71, 75, 74 and 82 were selected from these groups. Item 85 was removed due to not being related to NCDs’ perceived risk. Moreover, the experts added one more item for the perceived benefit construct. The items that are specific and not similar to other items in the respective domains remained in the questionnaire.

The second Delphi panel achieved a 51-item questionnaire (ten items in the susceptibility construct, six items in the severity construct, seven items in the benefit construct, 11 items in the barrier construct, nine items in the self-efficacy construct, and eight items in the behavioral change intention constructs) assuring content validity by the experts (S3 Table). Our experts also revised and edited the wording and sequencing of the items to make sure understanding.

We conducted a pre-test to assess the clarity and readability of the developed 51-item questionnaire among 15 people 25–60 years of age, representing both genders and from the middle to graduate education levels. During pre-testing, some participants gave suggestions to change question items to spoken patterns instead of sentence patterns and some words of question items to clarify and understand. We modified the items’ wording according to pre-testing results and suggestions from these participants to assure face validity. These changes were not much influenced to the original items’ intrinsic meaning; however, these changes improved the questionnaire’s clarity and accuracy in data collection.

#### Questionnaire validation

We ran univariate item analysis using collected data from 150 participants, and all the items’ means were from a minimum of 1.9 to a maximum of 3.5, and standard deviations were from 0.5 to 1. We also assessed the normality measure of the items, and the results showed no problems with skewness, but kurtosis existed among the items (S4 Table).

Before doing EFA, we assessed the sampling adequacy and sphericity assumptions. KMO measure indicated that a sample of 150 respondents was adequate (KMO = 0.783) to conduct EFA, and Bartlett’s test of sphericity rejected the null hypothesis of the correlation matrix was identical (P<0.001). Hence all assumptions were met for EFA.

**Fig 2:**
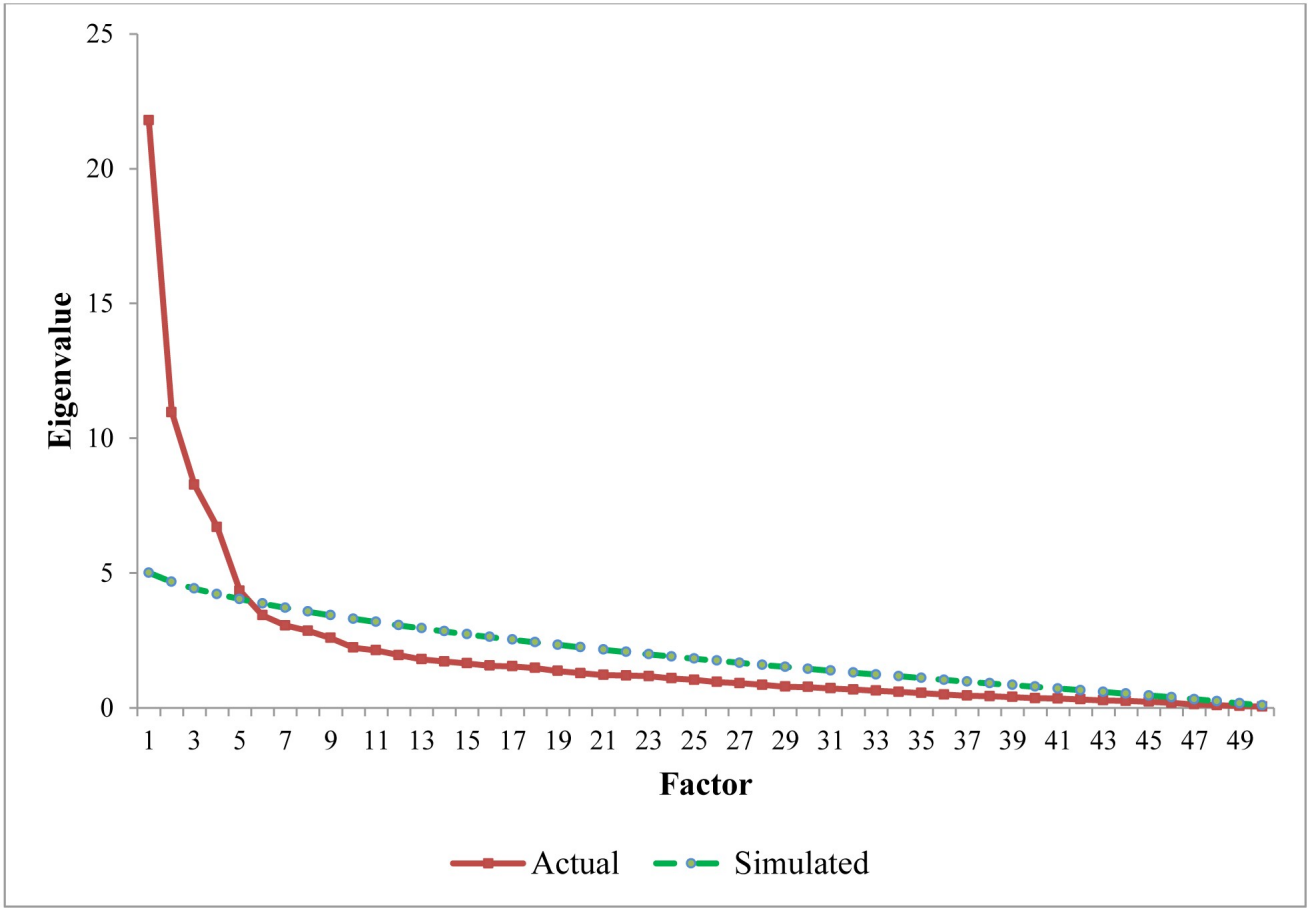
Parallel analysis scree plot.

The decision on how many factors need to be extracted was also a significant concern in factor analysis since both underfactoring and overfactoring cause problems. Underfactoring causes difficulties in interpreting the model due to substantial error, while overfactoring leads to developing unrealistic and complex theories. To avoid these problems, we used the parallel analysis method instead of using the Kaiser Criterion and the scree plot. We needed to extract the number of factors above the intersection point. Fig 2 showed the results of the parallel analysis using a parallel analysis scree plot. The parallel analysis revealed that the five-factor solution was the best for the EFA analysis since the simulated results cross the actual results between factors 5 and 6.

Moreover, we extracted one factor more and one factor less than the number provided by parallel analysis and observed the results to avoid under/overfactoring. The findings of four- and six-factor solutions were not good enough for theoretical interpretation, and some items were cross-loaded with more than one factor (See details in S5 Table). Therefore, we ran EFA with the five-factor model.

#### Exploratory factor analysis

We ran EFA using the maximum likelihood factor extraction method with the Promax rotation and Kaiser Normalization based on the polychoric correlation matrix. The first EFA output revealed that among 51 items, ten items loaded in factor 1, nine items loaded in factor 2, nine items in factor 3, eight items in factor 4, and seven items in factor 5. Among these items, items sus1 and sus2 cross-loaded in factors 3 and 5; and item intent6 cross-loaded in factors 1 and 4 (S6 Table—Pattern matrix 1). We removed the cross-loading items one after another and repeated EFA to obtain clean and theoretically meaningful results. First, we removed sus2 since the difference between two cross-loading items was lowest and reanalyzed EFA again. Then we removed sus1 and reran EFA. During this analysis, the previously unloaded item sus4 was cross-loaded between factors 2 and 5 with the difference loading 0.075; therefore, we removed this item and reanalyzed it again. After the removal of intent6, there was no more cross-loading. Still, two barrier items (bar1, bar2) loaded together with behavioral change items and one behavioral change item (intent8) with barrier items. We reran EFA by removing these items one by one to get reasonable and theoretically interpretable constructs. After these steps, there were eight self-efficacy items in factor 1, six benefit items and two severity items in factor 2, eight barrier items in factor 3, five behavioral change items in factor 4, and; five susceptibility items in factor 5. We named the factors according to loading items, i.e., Perceived self-efficacy (PerEffi) for factor 1, Perceived benefit (PerBene) for factor 2, Behavioral change intention (PerIntent) for factor 3, Perceived susceptibility (PerSus) for factor 4, and Perceived barrier (PerBar) for factor 5 (S6 Table—Pattern matrix 2).

We assessed each factor’s reliability using Cronbach’s alpha and removed the items that affected the reliability of factors. For the PerSus factor, the alpha value for five items was 0.774, and removing item sus3 increased alpha to 0.792. For the PerBene factor, the alpha for eight items was only 0.670, and removal of items bene1, seve3, and seve4 increased alpha to 0.831. The alpha value of PerBar was 0.757 using eight barrier items, and that of PerEffi was 0.837 for eight efficacy items. The PerIntent factor’s reliability was 0.846 for five behavioral change intention items. We ran the EFA again and again for the removal of every item for reliability reasons. This study’s main objective was to develop and validate the questionnaire using Confirmatory factor analysis (Structural Equation Modeling technique); hence, we included the items with loading greater than or equal to 0.5 in the final EFA model.

The final EFA model constituted a five-factor solution with twenty-two high-loading items. Six efficacy items were loaded together to the PerEffi factor, which accounted for 23.4% of the data variance with reliability alpha 0.834. Five benefit items were highly loaded to the PerBene factor and accounted for 11% of the total variance with alpha 0.831. Four behavioral change intention items were loaded to the PerIntent factor with reliability alpha 0.854 and accounted for 9.9% of the total variance. Four susceptibility items were loaded strongly to the PerSus factor, which accounted for 5.3% of the data variance with reliability alpha 0.792. Only three barrier items were loaded to the PerBar factor with the reliability of 0.683, and this factor accounted for 4% of the total variance. The five-factor model of EFA accounted for more than 50% of the total variance. The average factor loading of each factor was greater than or equal to 0.65, and this finding pointed out that the convergent validity of each factor was satisfactory.

All the factors’ reliabilities (Cronbach’s α) exceeded 0.7 except the PerBar factor, which has a reliability of nearly 0.7. These findings revealed that all factors’ reliabilities were satisfied to conduct CFA for the validation process of the 22-item questionnaire developed by EFA (See details in Table 1).

**Table 1:** Factor loading results and internal reliability of the factors of the final EFA model (22-item questionnaire).

Item	Factor					Communalities
	PerEffi	PerBene	PerIntent	PerSus	PerBar	
effi7	**.809**	-.124	.055	-.067	.020	.624
effi8	**.742**	.170	-.118	-.105	.039	.578
effi9	**.686**	-.069	.141	-.041	-.050	.535
effi2	**.664**	-.072	.055	.074	-.111	.500
effi3	**.558**	-.020	.098	.122	-.022	.371
effi6	**.535**	.250	-.137	.069	.054	.363
bene3	.024	**.724**	.116	-.051	.140	.669
bene2	.051	**.720**	.026	-.081	.208	.617
bene7	.107	**.709**	-.177	.210	-.176	.488
bene5	-.088	**.646**	.114	-.014	-.135	.500
bene4	-.053	**.626**	.180	-.069	-.081	.554
intent3	.109	-.085	**.862**	.042	.026	.711
intent2	.163	.021	**.750**	.009	.063	.664
intent4	-.132	.142	**.689**	.079	-.143	.589
intent1	-.046	.143	**.658**	-.053	.048	.550
sus5	.023	-.053	.018	**.745**	.047	.570
sus6	-.096	-.060	.004	**.736**	.056	.569
sus8	.105	.038	-.093	**.691**	.036	.523
sus10	-.017	.074	.153	**.640**	-.027	.413
bar4	.028	-.070	.001	-.015	**.768**	.577
bar7	-.141	.089	.140	.072	**.619**	.458
bar5	.024	-.029	-.166	.081	**.553**	.383
**Cronbach’s α**	.834	.831	.854	.792	.683	
**% of variance**	23.405	11.004	9.994	5.258	4.005	
**Cumulative %**	23.405	34.409	44.403	49.662	53.667	

PerEffi = Perceive self-efficacy, PerBene = Perceived benefit, PerIntent = Perceived behavioural change intention, PerSus = Perceived susceptibility, PerBar = Perceived barrier

We assessed the factor correlation matrix of the final exploratory factor analysis to check the discriminant validity. There were both negative and positive correlations among the five factors. The largest negative correlation was between PerEffi and PerBar (-0.249), and the smallest negative correlation was between PerSus and PerBene (-0.053). The largest positive correlation was between PerBene and PerIntent (0.577), and the lowest positive correlation was between PerBene and PerBar (0.025). There were no correlation coefficients greater than 0.7; hence, the factors derived from EFA revealed adequate discriminant validity among the factors (See details in Table 2).

**Table 2:** Factor correlation matrix of the final EFA model.

**Factor**	**PerEffi**	**PerBene**	**PerIntent**	**PerSus**	**PerBar**
PerEffi	1.000				
PerBene	.280	1.000			
PerIntent	.290	.577	1.000		
PerSus	.064	-.053	-.140	1.000	
PerBar	-.249	.025	-.159	.136	1.000

We assessed the common method bias (bias due to using a single data collection method that may introduce systematic response bias and inflate or deflate the responses) using Harman’s single factor test. This test used the maximum likelihood method and was forced to extract only one factor; whether to assess a single factor contributes more than 50% of the total variance. The results showed that the single factor model accounted for only 25.5%, which means no problem with common method bias in this study (S7 Table).

#### Confirmatory factor analysis

To determine whether EFA proposed a five-factor model with the 22-item questionnaire that can assess the perceived risk of developing non-communicable diseases at the population level, we ran CFA using a different sample of 210 participants. The CFA initial model showed that one efficacy item (effi6 item) had low loading (0.35). Hence we ran the CFA five-factor model with only 21 items dropping effi6 item.

**Fig 3:**
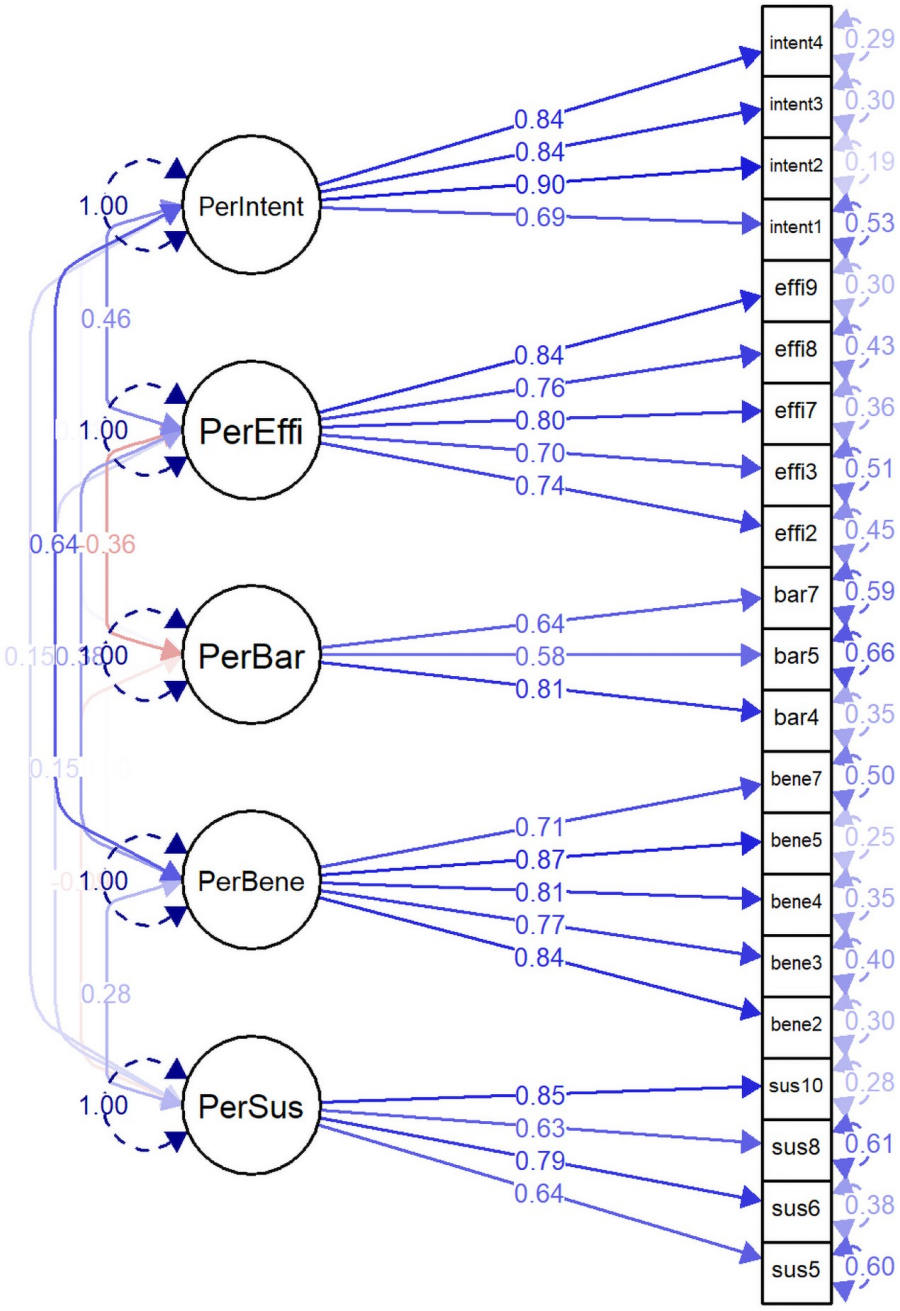
Perceived risk constructs of developing non-communicable diseases (Five-factor model).

We described the CFA five-factor model (NCD-PR5-21) with the SEM diagram (Fig 3). All latent factors were described in circles, and all indicator items were presented in rectangles. Bi-headed arrows represented the correlation between the latent variables, and single-headed arrows represented standardized loading of observed variables to latent factors. All loadings were ranged from 0.58 (bar5 item) to 0.87 (bene5 item), and the average factor loading of each factor was greater than 0.65. There was a moderate correlation between PerBene and PerIntent factors (0.64); however, all correlations between the latent factors were less than 0.7.

Standardized factor loadings of the five-factor model are presented in Table 3. All items were significantly loaded to each specific latent factor. We assessed the convergent validity and discriminant validity of NCD-PR5-21 during CFA based on the AVE values, which measured the amount of variance explained by a latent construct related to the error variance. The average variance extracted (AVE) values of PerSus, PerBene, PerEffi, and PerIntent factors were above 0.5, while those of PerBar were below 0.5, suggesting insufficient convergent validity of this factor. Although AVE values of PerBar were less than 0.5, factors specific item loadings were acceptable for convergent validity since there were no items with loading below 0.5. These items were also significant during CFA and measured the critical aspect of perceived risk in the respective domain. Hence we preserved this factor in the model.

**Table 3:** Standardized factor loadings, convergent validity, discriminant validity, reliability, and model fit indices of CFA (Five-factor model).

Items	Five-factor				
	PerSus	PerBene	PerBar	PerEffi	PerIntent
sus5	0.635				
sus6	0.785				
sus8	0.627				
sus10	0.849				
bene2		0.836			
bene3		0.774			
bene4		0.807			
bene5		0.868			
bene7		0.708			
bar4			0.808		
bar5			0.585		
bar7			0.638		
effi2				0.744	
effi3				0.697	
effi7				0.800	
effi8				0.758	
effi9				0.839	
intent1					0.687
intent2					0.899
intent3					0.839
intent4					0.841

			**Squared correlations (SC) among latent variables**				
**Factor**	**Ω**	**AVE**	**PerSus**	**PerBene**	**PerBar**	**PerEffi**	**PerIntent**
PerSus	0.769	0.533	0.533				
PerBene	0.816	0.640	(0.079)	0.640			
PerBar	0.652	0.467	(0.007)	(0.000)	0.467		
PerEffi	0.822	0.591	(0.021)	(0.144)	(0.133)	0.591	
PerIntent	0.800	0.673	(0.022)	(0.413)	(0.000)	(0.213)	0.673

**Model Fit indices**	**RMSEA**	**PCLOSE**	**CFI**	**TLI**	**SRMR**	**χ**^**2**^**/df**
	0.062 [0.051, 0.073]	0.039	0.924	0.910	0.071	1.79

Note: When AVE values > = SC values, there is no problem with discriminant validity. When AVE values > = 0.5 there is no problem with convergent validity, Ω = Raykov’s construct reliability (> = 0.7 is good reliability).

We also assessed the discriminant validity by comparing the square inter-factor correlation with AVE values. All square correlation values were lower than the AVE values of their respective factors; hence, there was no issue for the five-factor model’s discriminant validity. Among the five latent factors, only one factor (PerBar) had Raykov’s construct reliability (Ω) less than a minimum acceptable level of 0.7; however, its reliability was close to this level, i.e., 0.652. Regarding model fit indices of the five-factor model, the findings illustrated acceptable model-data-fit, i.e., RMSEA <0.08, PCLOSE = 0.039, relative chi-square <3, CFI = 0.924, TLI = 0.91 and, SRMR <0.08 (Table 3). Hence, CFA proved that the perceived risk of developing NCDs had underlying five latent factors. The 21-item questionnaire (NCD-PR5-21) has acceptable psychometric properties and model fitness to observed data (S8 Table).

**Fig 4:**
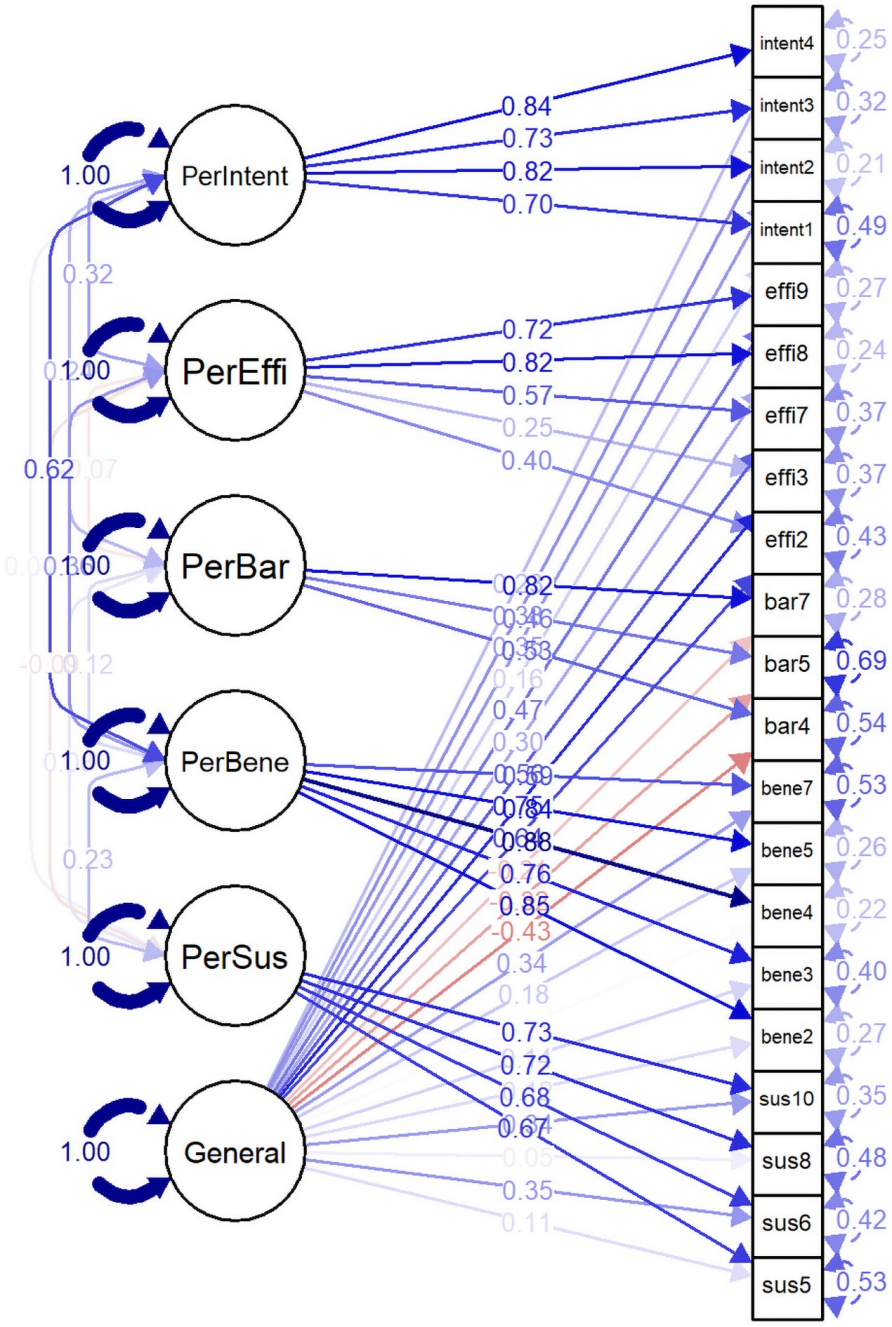
Perceived risk constructs of developing non-communicable diseases (Bifactor model).

**Fig 5:**
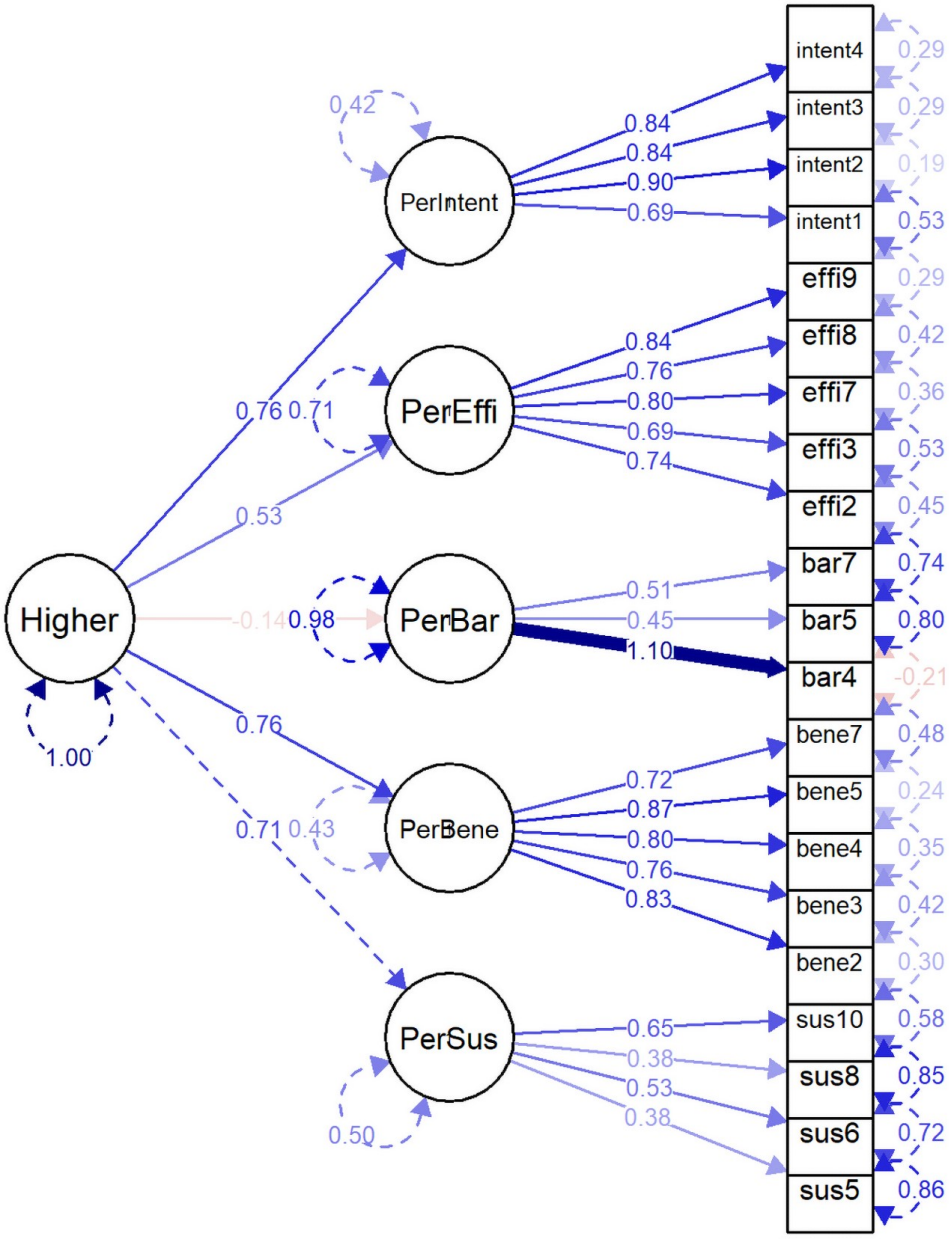
Perceived risk constructs of developing non-communicable diseases (Hierarchical model).

We also tested three alternative CFA models—single factor, bifactor, and hierarchical model. Standardized factor loadings of single-factor and bifactor models were reported in Table 4 and those of hierarchical model in Table 5. In the single-factor CFA model, all items were significantly loaded to the factor except for two barrier items (bar5 and bar7 items). We described the SEM diagram of the bifactor model of NCD-PR5-21 in Fig 4 and the hierarchical model in Fig 5. Although all items were significantly loaded to each specific latent factor in the bifactor model, six items (two susceptibility items, three benefit items, and one intention item) were not significant in the general factor. In the hierarchical model, all items were significantly loaded to respective factors, and all latent factors were significantly loaded to the second-order factor except for the PerBar factor.

**Table 4:** Standardized factor loadings of CFA for single-factor and bifactor models.

Items	Single-factor	Bifactor model					
	Factor	General	PerSus	PerBene	PerBar	PerEffi	PerIntent
sus5	0.157	0.109^ns^	0.674				
sus6	0.266	0.354	0.678				
sus8	0.156	0.051^ns^	0.718				
sus10	0.358	0.340	0.734				
bene2	0.701	0.115^ns^		0.847			
bene3	0.669	0.142^ns^		0.763			
bene4	0.662	0.011^ns^		0.882			
bene5	0.732	0.181		0.840			
bene7	0.638	0.338		0.593			
bar4	-0.186	-0.427			0.528		
bar5	-0.087^ns^	-0.318			0.458		
bar7	-0.012^ns^	-0.207			0.820		
effi2	0.498	0.636				0.405	
effi3	0.484	0.752				0.247	
effi7	0.564	0.558				0.568	
effi8	0.547	0.299				0.817	
effi9	0.583	0.427				0.716	
intent1	0.556	0.156^ns^					0.698
intent2	0.737	0.354					0.816
intent3	0.719	0.379					0.734
intent4	0.692	0.233					0.836

All loadings were significant at p < 0.05, except when mentioned.

^ns^, not significant.

**Table 5:** Standardized factor loadings of CFA for hierarchical model.

Items	Hierarchical model					
	PerSus	PerBene	PerBar	PerEffi	PerIntent	SecondOrder
sus5	0.378					
sus6	0.525					
sus8	0.382					
sus10	0.649					
bene2		0.834				
bene3		0.762				
bene4		0.804				
bene5		0.871				
bene7		0.721				
bar4			1.101			
bar5			0.450			
bar7			0.509			
effi2				0.740		
effi3				0.689		
effi7				0.800		
effi8				0.764		
effi9				0.844		
intent1					0.687	
intent2					0.898	
intent3					0.840	
intent4					0.842	
**Factors**						
PerSus						0.707
PerBene						0.758
PerBar						-0.136^ns^
PerEffi						0.534
PerIntent						0.765

All loadings were significant at p < 0.05, except when mentioned.

^ns^, not significant.

The model fit indices of three CFA models were reported in Table 6. CFI and TLI values of the bifactor model were the largest (0.954 and 0.938, respectively), and both RMSEA and SRMR were the smallest (0.051 and 0.054, respectively) compared to other models. This model’s PCLOSE was also not significant (0.418). Moreover, all model fit indices of the bifactor model were better than those of the five-factor model (See details in Table 3). Hence, the bifactor model is the best fit model for the observed data.

**Table 6:** Model fit indices of three measurement CFA models.

Measurement models	Model Fit indices					
	RMSEA	PCLOSE	CFI	TLI	SRMR	χ^2^/df
Single-factor model	0.153 [0.144, 0.161]	<0.001	0.509	0.454	0.156	5.86
Bifactor model with 5 specific factors	0.051 [0.038, 0.064]	0.418	0.954	0.938	0.054	1.55
Hierarchical model with 5 specific factors	0.092 [0.083, 0.102]	<0.001	0.825	0.802	0.105	2.76

To assess the questionnaire’s dimensionality (NCD-PR5-21), the ECV was calculated for the general construct and five specific constructs of the bifactor model, and the results were presented in Table 7. The general factor’s ECV value was very low, i.e., 20.4%, and it was strong evidence of the scale’s multidimensionality. We calculated the omega coefficient to evaluate the reliability of NCD-PR5-21 as a combination of general perceived risk and specific latent factors. In contrast, we calculated the omega hierarchical coefficient in assessing only general perceived risk or the specific factors that influenced its reliability (Table 7). The omega coefficient of the general factor (0.336) is below the cut-off value of 0.7, and other specific factors’ reliabilities are greater than 0.7, except for the PerBar factor (0.545) and PerEffi factor (0.516). The general factor’s omega hierarchical coefficient was only 0.194, while those of the specific factors were above the minimally acceptable level of 0.5 [34], except for the PerEffi factor. Construct replicability of the bifactor model was also assessed using H-index and the results were reported in Table 7. It was found that all factors revealed good construct replicability since the H-indices of general and specific factors were greater than 0.7.

**Table 7:** Indicators of dimensionality and reliability of the bifactor model of NCD-PR5-21.

	Bifactor model					
	General	PerSus	PerBene	PerBar	PerEffi	PerIntent
ECV	0.204	0.151	0.240	0.089	0.132	0.183
Ω	0.336	0.713	0.787	0.545	0.516	0.726
Ωh	0.194	0.709	0.786	0.515	0.412	0.707
H	0.802	0.796	0.911	0.729	0.791	0.865

ECV, explained common variance; Ω, omega (Raykov’s construct reliability); Ωh, omega hierarchical; H, H index (Construct replicability).

## Discussion

This study aimed to develop a questionnaire that can assess an individual’s perceived risk of getting non-communicable diseases among the Myanmar population. We followed the standard methodology of questionnaire development—item generation, scale development, and scale validation [35]. We specified the domain boundaries and generated items based on the constructs of HBM and adapting from the published validated questionnaires. First, we generated an 86-item question pool. Two rounds of Delphi panels ensured the content validity of the 51-item questionnaire, and pre-testing assured the cognitive debriefing for face validity. EFA removed the low loading items and perceived severity items, providing the five-factor model with a 22-item questionnaire.

We tested the psychometric properties of EFA proposed five-factor model of NCDs perceived risk questionnaire by conducting CFA using different samples. We removed one efficacy item during CFA due to low loading, and the rest of the analyses were done using a 21-item questionnaire. We run four different CFA models–(i) a single-factor, (ii) the EFA proposed five-factor, (iii) a bifactor (general factor and five specific factors), and (iv) a hierarchical model (Five specific factors and a second-order factor). The results showed that the bifactor model with a general perceived risk factor and five specific factors are superior to the rest models and showed good model fit indices. These findings proved that an individual’s perceived risk of getting NCDs depends on a general factor of perceived risk and five underlying specific latent constructs—how much they perceived on (i) disease susceptibility, (ii) the benefits of doing preventive activities, (iii) the barriers to exercise the preventive actions, (iv) capabilities to do preventive actions (efficacy), and (v) willingness of behavioral change intention to a healthy lifestyle.

The bifactor model confirmed the questionnaire’s multidimensional nature. All items were significantly loaded to each specific factor; however, some of the question items were not significantly loaded to the general perceived risk factor (See details in Table 4). The ECV of the general factor accounted only for twenty percent of the shared variance. This finding pointed out that although a general factor existed, it was not strong enough to assume the questionnaire’s unidimensional nature [31]. Moreover, it was evidence of the more critical nature of subscale scores over the total score in measuring an individual’s perceived risk of getting NCDs.

We calculated the omega and omega hierarchical indices to determine common sources of variance due to the general factor or specific factors. We also compare these indices to assess how much the general factor contributed to the total scores’ reliable variance. In our study, the difference between the omega and the omega hierarchical coefficient of the general factor was considerable (14.2%); however, for the specific latent factors, the differences were minimal, i.e., (0.4% for PerSus, 0.1% for PerBene, 3% for PerBar, 10.4% for PerEffi and 1.9% for PerIntent, respectively). Hence, the general factor contributed only 19.4% of the total score variance if we treated other specific factors’ variability as random error. This difference (14.2%) of the general factor was due to multidimensionality caused by the specific latent factors [32, 34]. There was no strong general perceived risk factor for getting NCDs, despite the observed data was multidimensional in nature.

Among PerBar and PerEffi items, effi2 and effi3 items’ loadings were higher in the general factor than their specific factor, i.e., (0.636 vs. 0.405 and 0.752 vs. 0.247, respectively), suggesting that these items are more likely to measure the general perceived risk factor. Moreover, bar4, bar5, and effi7 items’ loadings were similar for general and specific factors, i.e., (-0.427 vs. 0.528, -0.318 vs. 0.458 and 0.558 vs. 0.568, respectively), again indicating that these factors measured both general and specific factors equally. As a result, the reliabilities of PerBar and PerEffi factors were below the acceptable level. However, all factor-specific items were significantly loaded to their specific factors, and the average factor loadings of these factors were nearly 0.6 (Table 4). Apart from this, all factors’ H indices (construct replicability) were above the minimum acceptable level (Table 7) [32]. Hence, all latent factors were well identified by indicator variables, and NCD-PR5-21 shows the stability of latent constructs across the different studies.

Whether these five specific factors measure a single latent variable in measuring an individual’s perceived risk, we also ran the CFA hierarchical model. All question items were significantly loaded to respective factors, and all factors, except PerBar, were significantly loaded to the second-order factor (Table 5). However, the model fit indices of this model were poor, and these findings pointed out that there was no second-order factor in measuring an individual’s risk perception on getting NCDs.

Since we believe there might be some extent of correlation among the underlying factors of perceived risk, we used Promax rotation, one of the oblique rotation methods, which allowed us to correlate the extracted factors. EFA results showed a significant positive correlation of PerBene with PerEffi and PerIntent constructs (Table 2). If people perceive the benefit of preventive interventions, they are more confident and willing to change their risk behavior [36]. Moreover, PerEffi was negatively correlated with the PerBar construct so that people with less perceived barriers for recommended actions are also more confident to adopt healthy lifestyles [37].

We dropped the severity items during EFA for many reasons. Since the parallel analysis provided a five-factor model, one construct has no chance to be selected. Second, when we ran step by step EFA for item purification, these severity items were cross-loaded to more than one factor; hence, we removed them. Third, we assessed the internal consistency of constructs and removed some of these items to increase the items’ reliability. These severity items could not strongly correlate to form a factor like other latent factors (S5 & S6 Tables). One possible explanation for dropping severity items during EFA was that the developed items had no intrinsic ability to capture disease severity perception due to bias wording or ambiguous wording. Another reason was that the participants had no known NCDs; hence, they failed to perceive NCDs’ severity. Myanmar people might have different risk perceptions of NCDs’ disease severity due to various contextual and cultural factors, possibly failing to perceive the NCD disease severity. Moreover, the occurrence of NCDs’ adverse consequences after long-duration was another possible reason for failure to perceive disease severity as a construct. One of the meta-analysis studies, which accessed the viability of the HBM, also pointed out that perceived severity was the least powerful predictor for intended behavioral change among the six constructs [15].

This study does not intend to estimate the risk factors and the prevalence of NCDs. Instead, the study aims to develop a validated questionnaire to assess the risk perception of NCD. Hence the study’s findings are not useful for policy implications. However, the developed questionnaire can be used in the NCD clinics and in the community to identify high-risk groups, i.e., low perceived risk but high actual risk, especially for CVD. They are less likely to take preventive behavior and regular medical care; thus, they have higher chances of experiencing adverse health outcomes. Moreover, the questionnaire might provide individualized specific health messages according to their risk perception level for adopting healthy lifestyles and better treatment compliance, which will prevent them from the occurrence of adverse health outcomes.

### Strengths and limitations of the study

To the best of our knowledge, this study is the first study that developed and validated a questionnaire assessing an individual’s perceived risk of developing NCDs in Myanmar. As a rule of thumb, ten respondents for one scale item should be selected to obtain an adequate sample size. Our study included only 150 respondents for 51 items during the EFA; hence, the model might be underestimated. To check this possibility, we conducted an EFA again using all 360 respondents, and the results revealed the same five-factor model with very similar items (See details in S9 Table). Moreover, we checked sample size adequacy using the KMO statistic, and the result showed that 150 respondents were adequate for EFA analysis since the KMO value was greater than 0.7.

Our study also failed to assess the criterion-related validity due to the following reasons. Comparing risk perception scores between respondents with and without NCDs was impossible since we selected the participants with no known NCDs. No standardized tool is currently available to use as a gold standard to assess the individual’s perceived risk of NCDs. Hence, we could not assess the degree to which the scales of this study relate to other similar indicators and determine whether the sub-scales discriminate very well from different scales. Some items with loading less than 0.65 in CFA lowered the absolute model fit indices. These findings demonstrated that NCD-PR5-21 had limitations.

## Conclusion

The developed 21-item questionnaire (NCD-PR5-21) was valid and reliable to assess the general perceived risk construct and five specific constructs (susceptibility, benefit, barrier, self-efficacy, and behavioral change intention) of the perceived risk of getting four major NCDs among the Myanmar population. Further research is warranted to confirm the questionnaire’s reliability and validity and reproduce the factors that determined NCDs’ perceived risk with the larger sample to generalize the study’s findings. The questionnaire also needs to be tested the utility of a mismatch between risk perception and current risk; and individualized counseling for behavioral change communication.

## Supporting information

S1 Table Items pool for developing questionnaires for perceived risk on NCDs. (DOCX)

S2 Table Frequency distribution of background characteristics of participants by types of analysis. (DOCX)

S3 Table Development of the 51-item questionnaire. (DOCX)

S4 Table Univariate analysis of items that included in EFA analysis. (DOCX)

S5 Table Exploratory factor analysis for the six-factor solution and four-factor solution. (DOCX)

S6 Table Exploratory factor analysis—Initial step & factor naming step. (DOCX)

S7 Table Harman’s single factor test to check for common method bias. (DOCX)

S8 Table Validated final 21-item questionnaire (NCD-PR5-21). (DOCX)

S9 Table Factor loading results and internal reliability of the factors of the final EFA model using all 360 participants. (DOCX)

S1 Data (XLSX)

S2 Data (XLSX)
