# Supplementary material for: Developing and validating a questionnaire to assess an individual’s perceived risk of four major non-communicable diseases in Myanmar
Source: PLoS One. 2021 Apr 27;16(4):e0234281. doi: 10.1371/journal.pone.0234281 (PMC8078785; doi:10.1371/journal.pone.0234281)
Supplement: S1 Table — (DOCX) [file pone.0234281.s001.docx]

**S1 Table. Items pool for developing questionnaires for perceived risk on NCDs (Items were adapted and adopted from article-BMJ Open 2017;7:e014413. doi:10.1136)**

| Perceived vulnerability/  susceptibility | 1. There is a possibility that I will have NCDs. |
| --- | --- |
|  | 2. I have very little chance to get NCDs during the next 10 years. |
|  | 3. I have a high chance of getting NCDs because of my past behaviors. |
|  | 4. I feel sure that I will have NCDs. |
|  | 5. Healthy lifestyle habits are unattainable. |
|  | 6. It is likely that I will get one type of NCDs. |
|  | 7. I am at risk of having a NCD. |
|  | 8. It is possible that I will have a NCD. |
|  | 9. I am not doing any unhealthy lifestyle. |
|  | 10. I am too young to suffer from NCDs. |
|  | 11. People like me do not get a NCD. |
|  | 12. I am so healthy that I won’t suffer from NCDs. |
|  | 13. I am not worried that I might have a NCD. |
|  | 14. People of age 30 and below are too young to have a NCD. |
|  | 15. People of age 40 and below are too young to have a NCD. |
|  | 16. People of age 50 and below are too young to have a NCD. |
|  | 17. My lifestyle habits do not put me at risk for having a NCD. |
|  | 18. No matter what I do, if I am going to have a NCD, I will have one. |
|  | 19. People who do not have NCDs are just plain lucky. |
|  | 20. The exact causes of NCDs are unknown. |
|  | 21. It is likely that I will suffer from a NCD in the future. |
|  | 22. There is greater possibility that I would suffer from NCDs a few years later. |
|  | 23. Having a NCD is currently a possibility for me. |
|  | 24. I feel I will suffer from a NCD sometime during my life. |
|  | 25. I am concerned about the likelihood of having a NCD in the near future. |
| Perceived severity | 26. Having a NCD will have major effects on my life and family |
|  | 27. Having a NCD will not have major effects on my work and income |
|  | 28. Having a NCD will cripple me |
|  | 29. Having a NCD will not change my outlook |
|  | 30. Thought of having NCD scares me |
|  | 31. NCDs such as Heart attacks and strokes are always fatal. |
|  | 32. Having a NCD will threaten my relationship with my significant other. |
|  | 33. My whole life would change if I had a NCD e.g. heart attack or stroke. |
|  | 34. Having a NCD such as heart attack or stroke would have a very bad effect on my sex life. |
| Perceived benefits | 35. Increasing my exercise will decrease my chances of having a NCD. |
|  | 36. Eating a healthy diet will decrease my chance of having a NCD. |
|  | 37. Stopping smoking will reduce my chance of having NCDs. |
|  | 38. When I exercise I am doing something good for myself. |
|  | 39. When I eat healthy I am doing something good for myself. |
|  | 40. Cutting down on alcohol will decrease my chances of having NCDs. |
|  | 41. Not having NCDs is beneficial. |
|  | 42. Regular health check-ups will detect NCDs early. |
|  | 43. Regular health check-ups are not beneficial. |
| Perceived barriers | 44. Very little can be done to prevent NCDs |
|  | 45. Health check-ups are expensive |
|  | 46. Health check-ups are time-consuming |
|  | 47. It is embarrassing to go for health check-ups |
|  | 48. Treatment will not be effective in curing NCDs |
|  | 49. I do not know appropriate exercises to perform to reduce my risk of developing NCDs. |
|  | 50. I do not know the recommended drinking limits for men or women. |
|  | 51. I do not have time to exercise for 30 minutes a day on most days of the week. |
|  | 52. I do not know what is considered a healthy diet that would prevent me from developing NCDs. |
|  | 53. I will not have energy if I stop smoking. |
|  | 54. I cannot afford to buy healthy foods. |
|  | 55. I have other problems more important than worrying about NCDs. |
| Self-efficacy | 56. How confident are you to prevent NCDs? |
|  | 57. How confident are you to attend health assessments to prevent NCDs? |
|  | 58. How confident are you to have information to prevent NCDs? |
|  | 59. How confident are you to able to actively work on a healthy lifestyle to prevent NCDs? |
|  | 60. How confident are you to do to reduce my chances of getting NCDs? |
|  | 61. How confident are you that you know or can control the risks of having a heart attack or stroke? |
|  | 62. How confident are you that you know or can maintain a healthy weight by exercising regularly? |
|  | 63. How confident are you that you know or can stop smoking if you want to? |
|  | 64. How confident are you that you know or can consume less alcohol? |
|  | 65. How confident are you that you know or can control your blood pressure and/or cholesterol levels by taking your prescribed medications? |
|  | 66. How confident are you that you know or can eat a healthy and balanced diet? |
| Intention to Change Behavior or Cues to Action | 67. I want to stop smoking (if you do smoke). |
|  | 68. I intend to maintain a healthy weight. |
|  | 69. I intend to be physically active within two months. |
|  | 70. I expect to maintain a healthy weight. |
|  | 71. I want to be physically active. |
|  | 72. I intend to eat a healthy and balanced diet within two months. |
|  | 73. I expect to stop smoking (if you do smoke). |
|  | 74. I want to cut down on alcohol. |
|  | 75. I want to maintain a healthy and balanced diet. |
|  | 76. I intend to stop smoking within two months (if you do smoke). |
|  | 77. I expect to eat a healthy and balanced diet. |
|  | 78. I intend to cut down on alcohol in the next two months. |
|  | 79. I expect to be physically active. |
|  | 80. I expect to cut down on alcohol. |
|  | 81. I want to eat a healthy and balanced diet. |
|  | 82. I expect to take my medication to control my blood pressure and/or cholesterol |
|  | 83. I want to take my medication to control my blood pressure and/or cholesterol |
|  | 84. I intend to take my medication to control my blood pressure and/or cholesterol within two months |
|  | 85. I always follow medical orders to benefit my health |
|  | 86. I cannot make efforts to improve my health |
