# Supplementary material for: Developing and validating a questionnaire to assess an individual’s perceived risk of four major non-communicable diseases in Myanmar
Source: PLoS One. 2021 Apr 27;16(4):e0234281. doi: 10.1371/journal.pone.0234281 (PMC8078785; doi:10.1371/journal.pone.0234281)
Supplement: S2 Table — (DOCX) [file pone.0234281.s002.docx]

**S2 Table. Frequency distribution of background characteristics of participants by types of analysis**

| **Background characteristics** | **EFA (n=150)** | **CFA (n=210)** | **All (n=360)** |
| --- | --- | --- | --- |
| Age group (Years)  ≤ 40  >40 | 106 (70.7)  44 (29.3) | 133 (63.3)  77 (36.7) | 239 (66.4)  121 (33.6) |
| Sex  Male  Female | 40 (26.7)  110 (73.3) | 55 (26.2)  155 (73.8) | 95 (26.4)  265 (73.6) |
| Education*  No Formal education  Primary  Middle  High  University/College student  Graduate/Post graduate | 0 (0.0)  2 (1.3)  17 (11.3)  18 (12.0)  18 (12.0)  95 (63.3) | 1 (0.5)  6 (2.9)  15 (7.1)  21 (10.0)  15 (7.1)  150 (71.8) | 1 (0.3)  8 (2.2)  32 (8.9)  39 (10.8)  33 (9.2)  245 (68.1) |
| Occupation*  Dependent/housewife/student  Manual worker  Government staff  Company staff  Own business  Professional | 3 (2.0)  4 (2.7)  113 (75.3)  18 (12.0)  10 (6.7)  1 (0.7) | 12 (5.7)  17 (8.1)  103 (49.0)  50 (23.8)  23 (11.0)  2 (1.0) | 15 (4.2)  21 (5.8)  216 (60.0)  68 (18.9)  33 (9.2)  3 (0.8) |

* Missing values present.
