# Supplementary material for: Developing and validating a questionnaire to assess an individual’s perceived risk of four major non-communicable diseases in Myanmar
Source: PLoS One. 2021 Apr 27;16(4):e0234281. doi: 10.1371/journal.pone.0234281 (PMC8078785; doi:10.1371/journal.pone.0234281)
Supplement: S3 Table — (DOCX) [file pone.0234281.s003.docx]

**S3 Table. Development of the 51-item questionnaire**

| 1. Strongly disagree 2. Disagree 3. Agree 4. Strongly agree | | | | | | | | |
| --- | --- | --- | --- | --- | --- | --- | --- | --- |
| **Subscale** | **Item** | | | | **Answer** | | | |
| I. Perceived vulnerability/susceptibility | **Sus_1**. I am too young to suffer from NCDs. | | | | 1 | 2 | 3 | 4 |
|  | **Sus_2**. I am so healthy that I won’t suffer from NCDs. | | | | 1 | 2 | 3 | 4 |
|  | **Sus_3**. There is a greater possibility that I would suffer from NCDs a few years later. | | | | 1 | 2 | 3 | 4 |
|  | **Sus_4**. I may suffer from NCDs if I don’t care about my lifestyle. | | | | 1 | 2 | 3 | 4 |
|  | **Sus_5**. I would likely suffer from any of the NCDs. | | | | 1 | 2 | 3 | 4 |
|  | **Sus_6**. It is almost sure that I would suffer from NCDs. | | | | 1 | 2 | 3 | 4 |
|  | **Sus_7**. People aged 40 and below are too young to have an NCD. | | | | 1 | 2 | 3 | 4 |
|  | **Sus_8**. There is a possibility that I would suffer from NCDs at this moment. | | | | 1 | 2 | 3 | 4 |
|  | **Sus_9**. No matter what I do, if I am going to have an NCD, I will have one. | | | | 1 | 2 | 3 | 4 |
|  | **Sus_10**. I feel I will suffer from an NCD sometime during my life. | | | | 1 | 2 | 3 | 4 |
| II. Perceived severity | **Seve_1**. There will not be a significant impact on my family if I suffer from NCDs. | | | | 1 | 2 | 3 | 4 |
|  | **Seve_2**. I think that having any NCDs might have a severe impact on my sexuality. | | | | 1 | 2 | 3 | 4 |
|  | **Seve_3**. If I suffer from NCDs, it will make me disabled. | | | | 1 | 2 | 3 | 4 |
|  | **Seve_4**. If I suffer from NCDs, it will have a profound impact on my job and income. | | | | 1 | 2 | 3 | 4 |
|  | **Seve_5**. I am scared of the thought of having NCDs. | | | | 1 | 2 | 3 | 4 |
|  | **Seve_6**. NCDs are not deadly diseases. | | | | 1 | 2 | 3 | 4 |
| III. Perceived benefits | **Bene_1**. Quitting smoking/betel chewing will not reduce the possibility of getting NCDs. | | | | 1 | 2 | 3 | 4 |
|  | **Bene_2.** Doing physical exercises can prevent NCDs. | | | | 1 | 2 | 3 | 4 |
|  | **Bene_3.** Eating a healthy diet can prevent NCDs. | | | | 1 | 2 | 3 | 4 |
|  | **Bene_4.** The reduction of drinking an excessive amount of alcohol can prevent NCDs. | | | | 1 | 2 | 3 | 4 |
|  | **Bene_5.** The regular medical checkup is necessary to get an early diagnosis of NCD. | | | | 1 | 2 | 3 | 4 |
|  | **Bene_6.** Free from NCDs is beneficial to my family and me. | | | | 1 | 2 | 3 | 4 |
|  | **Bene_7.** Living stress-free can prevent NCDs. | | | | 1 | 2 | 3 | 4 |
| IV. Perceived barriers | **Bar_1.** Medical checkup costs a lot of money. / It is costly to do a medical checkup. | | | | 1 | 2 | 3 | 4 |
|  | **Bar_2.** It takes time to do a medical checkup. / It is time-consuming to do a medical checkup. | | | | 1 | 2 | 3 | 4 |
|  | **Bar_3.** I don’t do medical checkups because I’m afraid of being diagnosed with diseases. | | | | 1 | 2 | 3 | 4 |
|  | **Bar_4.** I don’t know about the suitable physical exercises which would help reduce the possibility of suffering from NCDs. | | | | 1 | 2 | 3 | 4 |
|  | **Bar_5.** On most of the days of a week, I don’t have time to do a physical activity of 30 minutes a day. | | | | 1 | 2 | 3 | 4 |
|  | **Bar_6.** I do not know the recommended drinking limits for men or women. | | | | 1 | 2 | 3 | 4 |
|  | **Bar_7.** I don’t know which type of diet can prevent NCDs. | | | | 1 | 2 | 3 | 4 |
|  | **Bar_8.** I cannot afford to buy a healthy diet. | | | | 1 | 2 | 3 | 4 |
|  | **Bar_9.** If someone who is addicted to smoking/ betel chewing quits, he/she will not be active anymore. | | | | 1 | 2 | 3 | 4 |
|  | **Bar_10.** There are very few methods to prevent NCDs. | | | | 1 | 2 | 3 | 4 |
|  | **Bar_11.** I have some other things which are more critical than it is to worry about NCDs. | | | | 1 | 2 | 3 | 4 |
| 1. Not at all confident 2. Somewhat confident 3. Moderately confident 4.Completely confident | | | | | | | | |
| V. Self-efficacy | **Effi_1.** How much extent do you believe in yourself to take the medical checkup to prevent NCDs? | | | | 1 | 2 | 3 | 4 |
|  | **Effi_2.** How much extent do you believe in yourself to live healthily to prevent NCDs? | | | | 1 | 2 | 3 | 4 |
|  | **Effi_3.** How much extent do you believe in yourself to maintain suitable body weight by doing regular physical activity to prevent NCDs? | | | | 1 | 2 | 3 | 4 |
|  | **Effi_4.** Supposed you are addicted to smoking/betel chewing; how much extent do you believe if you can quit smoking/ betel chewing? | | | | 1 | 2 | 3 | 4 |
|  | **Effi_5.** How much extent do you believe in someone who is addicted to alcohol to reduce alcohol intake to prevent NCDs? | | | | 1 | 2 | 3 | 4 |
|  | **Effi_6.** How much extent do you believe in yourself to take the prescribed drugs to prevent the complications of NCDs? | | | | 1 | 2 | 3 | 4 |
|  | **Effi_7.** How much extent do you believe in yourself to eat a healthy diet only? | | | | 1 | 2 | 3 | 4 |
|  | **Effi_8.** How much extent do you believe in yourself to reduce the risk of NCDs? | | | | 1 | 2 | 3 | 4 |
|  | **Effi_9.** How much extent do you believe in yourself to practice healthy habits? | | | | 1 | 2 | 3 | 4 |
| 1. Strongly disagree | | 1. Disagree | 1. Agree | 1. Strongly agree | | | | |
| VI. Intention to change behavior or cues to action | **Intent_1.** Supposed I am addicted to smoking/betel chewing; I will quit smoking/betel chewing to prevent NCDs. | | | | 1 | 2 | 3 | 4 |
|  | **Intent_2.** I will maintain suitable bodyweight to prevent NCDs. | | | | 1 | 2 | 3 | 4 |
|  | **Intent_3.** I will do physical activities actively to prevent NCDs. | | | | 1 | 2 | 3 | 4 |
|  | **Intent_4.** I will reduce (or) quit alcohol drinking to prevent NCDs. | | | | 1 | 2 | 3 | 4 |
|  | **Intent_5.** I will change to eat a healthy balanced diet (or) maintain an eating healthy balanced diet to prevent NCDs. | | | | 1 | 2 | 3 | 4 |
|  | **Intent_6.** I will take regular medical checkups to prevent NCDs. | | | | 1 | 2 | 3 | 4 |
|  | **Intent_7.** I will take prescribed drugs regularly if I have either hypertension or hyperlipidemia or both. | | | | 1 | 2 | 3 | 4 |
|  | **Intent_8.** I am not able to make efforts to prevent NCDs. | | | | 1 | 2 | 3 | 4 |
