# Supplementary material for: Developing and validating a questionnaire to assess an individual’s perceived risk of four major non-communicable diseases in Myanmar
Source: PLoS One. 2021 Apr 27;16(4):e0234281. doi: 10.1371/journal.pone.0234281 (PMC8078785; doi:10.1371/journal.pone.0234281)
Supplement: S4 Table — (DOCX) [file pone.0234281.s004.docx]

**S4 Table. Univariate analysis of items that included in EFA analysis**

| **Item code** | **Items** | | | | **Mean** | | **SD** | **N** |
| --- | --- | --- | --- | --- | --- | --- | --- | --- |
| sus_1 | I am too young to suffer from NCDs. | | | | 2.1 | | .8 | 150 |
| sus_2 | I am so healthy that I won’t suffer from NCDs. | | | | 2.1 | | .8 | 150 |
| sus_3 | There is a greater possibility that I would suffer from  NCDs a few years later. | | | | 2.5 | | .7 | 150 |
| sus_4 | I may suffer from NCDs if I don’t care about my lifestyle. | | | | 3.2 | | .6 | 150 |
| sus_5 | It is likely that I would suffer from any of the NCDs. | | | | 2.8 | | .7 | 150 |
| sus_6 | It is almost certain that I would suffer from NCDs. | | | | 2.2 | | .8 | 150 |
| sus_7 | It is too young for people aged 40 years and below to  suffer from NCDs. | | | | 2.1 | | .8 | 150 |
| sus_8 | There is a possibility that I would suffer from NCDs at this moment. | | | | 2.4 | | .8 | 150 |
| sus_9 | No matter how I live healthily, I might not be able to  avoid NCDs if I am destined to suffer from NCDs. | | | | 2.7 | | .9 | 150 |
| sus_10 | I will suffer from NCDs sometime in my life. | | | | 2.7 | | .7 | 150 |
| seve_1 | There will not be a great impact on my family if I  suffer from NCDs. | | | | 1.9 | | .7 | 150 |
| seve_2 | I think that having any NCDs might have a serious impact on my sexuality. | | | | 2.6 | | .7 | 150 |
| seve_3 | If I suffer from NCDs, it will make me disabled. | | | | 3.0 | | .7 | 150 |
| seve_4 | If I suffer from NCDs, it will have a profound impact on my job and income. | | | | 3.2 | | .6 | 150 |
| seve_5 | I am scared of the thought of having NCDs. | | | | 2.7 | | .9 | 150 |
| seve_6 | NCDs are not deadly diseases. | | | | 2.1 | | .7 | 150 |
| bene_1 | Quitting of smoking/betel chewing will not reduce the  possibility of getting NCDs. | | | | 2.0 | | .7 | 150 |
| bene_2 | Doing physical exercises can prevent NCDs. | | | | 3.2 | | .5 | 150 |
| bene_3 | Eating a healthy diet can prevent NCDs. | | | | 3.3 | | .5 | 150 |
| bene_4 | Reduction of drinking excessive amount of alcohol  can prevent NCDs. | | | | 3.1 | | .6 | 150 |
| bene_5 | Early diagnosis of NCDs can be achieved by regular medical checkups. | | | | 3.3 | | .5 | 150 |
| bene_6 | Free from NCDs is beneficial to my family and me. | | | | 3.4 | | .6 | 150 |
| bene_7 | Living stress-free can prevent NCDs. | | | | 3.1 | | .6 | 150 |
| bar_1 | Medical checkup costs a lot of money. / It is costly to do a medical checkup. | | | | 3.0 | | .8 | 150 |
| bar_2 | It takes time to do a medical checkup. / It is time-consuming to do a medical checkup. | | | | 3.1 | | .6 | 150 |
| bar_3 | I don’t do medical checkups because I’m afraid of being diagnosed with diseases. | | | | 2.0 | | .9 | 150 |
| bar_4 | I don’t know about the suitable physical exercises  which would help reduce the possibility of suffering  from NCDs. | | | | 2.3 | | .7 | 150 |
| bar_5 | On most of the days of a week, I don’t have time to do a physical activity of 30 minutes a day. | | | | 2.2 | | .7 | 150 |
| bar_6 | I do not know the recommended drinking limits for men or women. | | | | 2.9 | | .7 | 150 |
| bar_7 | I don’t know which type of diet can prevent NCDs. | | | | 2.4 | | .7 | 150 |
| bar_8 | I cannot afford to buy a healthy diet. | | | | 2.2 | | .8 | 150 |
| bar_9 | If someone who is addicted to smoking/ betel chewing  quits, he/she will not be active anymore. | | | | 2.2 | | .8 | 150 |
| bar_10 | There are very few methods to prevent NCDs. | | | | 1.8 | | .7 | 150 |
| bar_11 | I have some other more important things than it is to worry about NCDs. | | | | 2.4 | | .8 | 150 |
| effi_1 | How much extent do you believe in yourself to take  the medical checkup in order to prevent NCDs? | | | | 3.3 | | .8 | 150 |
| effi_2 | How much extent do you believe in yourself to live  healthily in order to prevent NCDs? | | | | 3.2 | | .7 | 150 |
| effi_3 | How much extent do you believe in yourself to  maintain suitable body weight by doing regular  physical activity in order to prevent NCDs? | | | | 3.3 | | .7 | 150 |
| effi_4 | Supposed someone who is addicted to smoking/betel  chewing has the willingness to prevent NCDs, how  much extent do you believe if he/she can quit  smoking/ betel chewing? | | | | 2.7 | | 1.0 | 150 |
| effi_5 | How much extent do you believe in someone who is  addicted to alcohol to reduce alcohol intake in order  to prevent NCDs? | | | | 2.3 | | 1.0 | 150 |
| effi_6 | How much extent do you believe in yourself to take  the prescribed drugs in order to prevent the  complications of NCDs? | | | | 3.1 | | .8 | 150 |
| effi_7 | How much extent do you believe in yourself to eat  a healthy diet only? | | | | 3.0 | | .8 | 150 |
| effi_8 | How much extent do you believe in yourself to reduce the risk of NCDs? | | | | 2.9 | | .7 | 150 |
| effi_9 | How much extent do you believe in yourself to practice healthy habits? | | | | 3.1 | | .7 | 150 |
| intent_1 | Supposed I am addicted to smoking/betel chewing; I will quit smoking/betel chewing in order to prevent NCDs. | | | | 3.0 | | .8 | 150 |
| intent_2 | I will maintain suitable bodyweight in order to prevent NCDs. | | | | 3.2 | | .6 | 150 |
| intent_3 | I will do physical activities actively in order to prevent NCDs. | | | | 3.1 | | .6 | 150 |
| intent_4 | I will reduce (or) quit alcohol drinking in order to prevent NCDs. | | | | 3.2 | | .8 | 150 |
| intent_5 | I will change to eat a healthy balanced diet (or) maintain an eating healthy balanced diet in order to prevent NCDs. | | | | 3.2 | | .5 | 150 |
| intent_6 | I will take regular medical checkups in order to prevent NCDs. | | | | 3.0 | | .8 | 150 |
| intent_7 | I will take prescribed drugs regularly if I am diagnosed with hypertension and/or hyperlipidemia. | | | | 3.5 | | .6 | 150 |
| intent_8 | I am not able to make efforts to prevent NCDs. | | | | 1.9 | | .8 | 150 |
| **Multivariate asymmetry skewness and kurtosis** | | | | | | | | |
|  | | Coefficient | Statistic | df | | P | | |
| Skewness | | 1207.045 | 30377.288 | 23426 | | 1.000 | | |
| Skewness corrected for small sample | | 1207.045 | 31004.346 | 23426 | | 1.000 | | |
| Kurtosis | | 2886.01 | 15.293 |  | | 0.0000** | | |
