# Supplementary material for: Developing and validating a questionnaire to assess an individual’s perceived risk of four major non-communicable diseases in Myanmar
Source: PLoS One. 2021 Apr 27;16(4):e0234281. doi: 10.1371/journal.pone.0234281 (PMC8078785; doi:10.1371/journal.pone.0234281)
Supplement: S5 Table — (DOCX) [file pone.0234281.s005.docx]

**S5 Table. Exploratory factor analysis for six-factor solution and four-factor solution**

| **Pattern matrix 3 (Six-factor solution)** | | | | | | |  | **Pattern matrix 4 (Four-factor solution)** | | | | |
| --- | --- | --- | --- | --- | --- | --- | --- | --- | --- | --- | --- | --- |
|  | Factor | | | | | |  |  | Factor | | | |
|  | 1 | 2 | 3 | 4 | 5 | 6 |  |  | 1 | 2 | 3 | 4 |
| effi_7 | 0.793 |  |  |  |  |  |  | bene_3 | 0.786 |  |  |  |
| effi_9 | 0.743 |  |  |  |  |  |  | bene_5 | 0.707 |  |  |  |
| effi_8 | 0.710 |  |  |  |  |  |  | bene_2 | 0.696 |  |  |  |
| effi_2 | 0.673 |  |  |  |  |  |  | bene_4 | 0.646 |  |  |  |
| effi_6 | 0.594 |  |  |  |  |  |  | bene_6 | 0.609 |  |  |  |
| effi_3 | 0.544 |  |  |  |  |  |  | intent_2 | 0.587 |  |  |  |
| effi_4 | 0.424 |  |  |  |  |  |  | intent_1 | 0.587 |  |  |  |
| effi_1 |  |  |  |  |  |  |  | intent_4 | 0.574 |  |  |  |
| effi_5 |  |  |  |  |  |  |  | intent_3 | 0.536 |  |  |  |
| bene_3 |  | 0.739 |  |  |  |  |  | seve_4 | 0.527 |  |  |  |
| bene_2 |  | 0.671 |  |  |  |  |  | sus_4 | 0.425 |  |  |  |
| seve_3 |  | 0.668 |  |  |  |  |  | bene_1 | -0.420 |  |  |  |
| bene_1 |  | -0.62 |  |  |  |  |  | bene_7 | 0.403 |  |  |  |
| seve_4 |  | 0.542 |  |  |  |  |  | bar_2 |  |  |  |  |
| bene_5 |  | 0.516 |  |  |  |  |  | bar_1 |  |  |  |  |
| bene_4 |  | 0.488 |  |  |  |  |  | seve_3 |  |  |  |  |
| seve_1 |  | -0.48 |  |  |  |  |  | bar_6 |  |  |  |  |
| bene_7 |  | 0.422 |  |  |  |  |  | seve_6 |  |  |  |  |
| seve_6 |  | -0.41 |  |  |  |  |  | seve_1 |  |  |  |  |
| bene_6 |  |  |  |  |  |  |  | intent_7 |  |  |  |  |
| bar_4 |  |  | 0.634 |  |  |  |  | seve_2 |  |  |  |  |
| bar_7 |  |  | 0.603 |  |  |  |  | effi_7 |  | 0.787 |  |  |
| bar_5 |  |  | 0.556 |  |  |  |  | effi_8 |  | 0.738 |  |  |
| bar_3 |  |  | 0.554 |  |  |  |  | effi_2 |  | 0.687 |  |  |
| bar_10 |  |  | 0.528 |  |  |  |  | effi_9 |  | 0.640 |  |  |
| bar_9 |  |  | 0.526 |  |  |  |  | effi_6 |  | 0.570 |  |  |
| bar_11 |  |  | 0.498 |  |  |  |  | effi_3 |  | 0.558 |  |  |
| bar_8 |  |  | 0.435 |  |  |  |  | effi_4 |  | 0.508 |  |  |
| bar_6 |  |  |  |  |  |  |  | intent_6 |  | 0.495 |  |  |
| intent_8 |  |  |  |  |  |  |  | effi_5 |  | 0.485 |  |  |
| intent_4 |  |  |  | 0.678 |  |  |  | effi_1 |  | 0.458 |  |  |
| intent_3 |  |  |  | 0.672 |  |  |  | intent_5 |  |  |  |  |
| intent_1 |  |  |  | 0.655 |  |  |  | bar_7 |  |  | 0.624 |  |
| intent_2 |  |  |  | 0.629 |  |  |  | bar_4 |  |  | 0.616 |  |
| bar_2 |  |  |  | 0.592 |  |  |  | bar_5 |  |  | 0.574 |  |
| intent_6 |  |  |  | 0.514 |  |  |  | bar_9 |  |  | 0.566 |  |
| bar_1 |  |  | 0.461 | 0.513 |  |  |  | bar_10 |  |  | 0.544 |  |
| intent_5 |  |  |  | 0.436 |  |  |  | bar_3 |  |  | 0.493 |  |
| intent_7 |  |  |  |  |  |  |  | intent_8 |  |  | 0.414 |  |
| seve_5 |  |  |  |  |  |  |  | bar_11 |  |  | 0.406 |  |
| sus_8 |  |  |  |  | 0.74 |  |  | bar_8 |  |  |  |  |
| sus_6 |  |  |  |  | 0.733 |  |  | sus_9 |  |  |  |  |
| sus_5 |  |  |  |  | 0.709 |  |  | sus_8 |  |  |  | 0.707 |
| sus_10 |  |  |  |  | 0.617 |  |  | sus_5 |  |  |  | 0.703 |
| sus_1 |  |  |  |  | -0.52 | 0.444 |  | sus_10 |  |  |  | 0.665 |
| sus_4 |  |  |  |  | 0.433 |  |  | sus_6 |  |  |  | 0.653 |
| sus_3 |  |  |  |  |  |  |  | sus_1 |  |  |  | -0.626 |
| sus_9 |  |  |  |  |  |  |  | sus_2 |  |  |  | -0.477 |
| seve_2 |  |  |  |  |  | -0.47 |  | sus_3 |  |  |  | 0.410 |
| sus_7 |  |  |  |  |  | 0.436 |  | sus_7 |  |  |  |  |
| sus_2 |  |  |  |  |  |  |  | seve_5 |  |  |  |  |
