# Supplementary material for: Developing and validating a questionnaire to assess an individual’s perceived risk of four major non-communicable diseases in Myanmar
Source: PLoS One. 2021 Apr 27;16(4):e0234281. doi: 10.1371/journal.pone.0234281 (PMC8078785; doi:10.1371/journal.pone.0234281)
Supplement: S6 Table — (DOCX) [file pone.0234281.s006.docx]

**S6 Table. Exploratory factor analysis – Initial step & Factor naming step**

| **Pattern Matrix – 1 (Initial step)** | | | | | |  | **Pattern Matrix – 2 (Factor naming step)** | | | | | |
| --- | --- | --- | --- | --- | --- | --- | --- | --- | --- | --- | --- | --- |
|  | Factor | | | | |  |  | Factor | | | | |
|  | 1 | 2 | 3 | 4 | 5 |  |  | PerEffi | PerBene | PerBar | PerIntent | PerSus |
| effi_7 | 0.776 |  |  |  |  |  | effi_8 | 0.785 |  |  |  |  |
| effi_8 | 0.750 |  |  |  |  |  | effi_7 | 0.746 |  |  |  |  |
| effi_2 | 0.669 |  |  |  |  |  | effi_2 | 0.673 |  |  |  |  |
| effi_9 | 0.638 |  |  |  |  |  | effi_9 | 0.640 |  |  |  |  |
| effi_6 | 0.605 |  |  |  |  |  | effi_6 | 0.585 |  |  |  |  |
| effi_3 | 0.549 |  |  |  |  |  | effi_3 | 0.524 |  |  |  |  |
| effi_4 | 0.500 |  |  |  |  |  | effi_4 | 0.486 |  |  |  |  |
| effi_5 | 0.463 |  |  |  |  |  | effi_5 | 0.452 |  |  |  |  |
| effi_1 | 0.425 |  |  |  |  |  | effi_1 |  |  |  |  |  |
| bene_3 |  | 0.699 |  |  |  |  | bene_3 |  | 0.775 |  |  |  |
| seve_3 |  | 0.659 |  |  |  |  | bene_2 |  | 0.733 |  |  |  |
| bene_2 |  | 0.634 |  |  |  |  | bene_5 |  | 0.637 |  |  |  |
| bene_1 |  | -0.604 |  |  |  |  | seve_3 |  | 0.573 |  |  |  |
| seve_4 |  | 0.508 |  |  |  |  | bene_7 |  | 0.545 |  |  |  |
| bene_5 |  | 0.475 |  |  |  |  | seve_4 |  | 0.532 |  |  |  |
| seve_1 |  | -0.465 |  |  |  |  | bene_1 |  | -0.525 |  |  |  |
| bene_4 |  | 0.418 |  |  |  |  | bene_4 |  | 0.477 |  |  |  |
| seve_6 |  | -0.414 |  |  |  |  | bene_6 |  |  |  |  |  |
| bene_6 |  |  |  |  |  |  | seve_2 |  |  |  |  |  |
| seve_2 |  |  |  |  |  |  | seve_6 |  |  |  |  |  |
| bene_7 |  |  |  |  |  |  | seve_1 |  |  |  |  |  |
| bar_7 |  |  | 0.610 |  |  |  | bar_7 |  |  | 0.656 |  |  |
| bar_4 |  |  | 0.605 |  |  |  | bar_4 |  |  | 0.654 |  |  |
| bar_9 |  |  | 0.565 |  |  |  | bar_10 |  |  | 0.594 |  |  |
| bar_5 |  |  | 0.565 |  |  |  | bar_5 |  |  | 0.572 |  |  |
| bar_10 |  |  | 0.540 |  |  |  | bar_9 |  |  | 0.499 |  |  |
| bar_3 |  |  | 0.493 |  |  |  | bar_3 |  |  | 0.472 |  |  |
| bar_11 |  |  | 0.415 |  |  |  | bar_11 |  |  | 0.453 |  |  |
| intent_8 |  |  |  |  |  |  | bar_8 |  |  | 0.422 |  |  |
| bar_8 |  |  |  |  |  |  | bar_6 |  |  |  |  |  |
| sus_9 |  |  |  |  |  |  | intent_7 |  |  |  |  |  |
| bar_6 |  |  |  |  |  |  | intent_3 |  |  |  | 0.919 |  |
| intent_4 |  |  |  | 0.718 |  |  | intent_2 |  |  |  | 0.749 |  |
| intent_1 |  |  |  | 0.682 |  |  | intent_1 |  |  |  | 0.738 |  |
| intent_3 |  |  |  | 0.673 |  |  | intent_4 |  |  |  | 0.712 |  |
| bar_2 |  |  |  | 0.630 |  |  | intent_5 |  |  |  | 0.443 |  |
| intent_2 |  |  |  | 0.608 |  |  | sus_10 |  |  |  |  | 0.703 |
| intent_6 | 0.426 |  |  | 0.538 |  |  | sus_5 |  |  |  |  | 0.693 |
| bar_1 |  |  |  | 0.497 |  |  | sus_8 |  |  |  |  | 0.691 |
| intent_5 |  |  |  | 0.415 |  |  | sus_6 |  |  |  |  | 0.670 |
| intent_7 |  |  |  |  |  |  | sus_3 |  |  |  |  | 0.419 |
| sus_8 |  |  |  |  | 0.705 |  | sus_7 |  |  |  |  |  |
| sus_5 |  |  |  |  | 0.692 |  | sus_9 |  |  |  |  |  |
| sus_1 |  |  | 0.408 |  | -0.660 |  | seve_5 |  |  |  |  |  |
| sus_10 |  |  |  |  | 0.657 |  | Extraction Method: Maximum Likelihood. | | | | | |
| sus_6 |  |  |  |  | 0.638 |  | Rotation Method: Promax with Kaiser Normalization. | | | | | |
| sus_2 |  |  | 0.410 |  | -0.506 |  | a. Rotation converged in 6 iterations. | | | | | |
| sus_3 |  |  |  |  | 0.410 |  |  |  |  |  |  |  |
| sus_7 |  |  |  |  |  |  |  |  |  |  |  |  |
| sus_4 |  |  |  |  |  |  |  |  |  |  |  |  |
| Seve_5 |  |  |  |  |  |  |  |  |  |  |  |  |
| Extraction Method: Maximum Likelihood. | | | | | |  |  |  |  |  |  |  |
| Rotation Method: Promax with Kaiser Normalization. | | | | | |  |  |  |  |  |  |  |
| a. Rotation converged in 6 iterations. | | | | | |  |  |  |  |  |  |  |
