# Supplementary material for: Developing and validating a questionnaire to assess an individual’s perceived risk of four major non-communicable diseases in Myanmar
Source: PLoS One. 2021 Apr 27;16(4):e0234281. doi: 10.1371/journal.pone.0234281 (PMC8078785; doi:10.1371/journal.pone.0234281)
Supplement: S7 Table — (DOCX) [file pone.0234281.s007.docx]

**S7 Table. Harman's single factor test to check for common method bias**

| Component | Initial Eigenvalues | | | Extraction Sums of Squared Loadings | | |
| --- | --- | --- | --- | --- | --- | --- |
|  | Total | % of Variance | Cumulative % | Total | % of Variance | Cumulative % |
| 1 | 5.606 | 25.480 | 25.480 | 5.606 | 25.480 | 25.480 |
| 2 | 2.892 | 13.145 | 38.625 |  |  |  |
| 3 | 2.660 | 12.093 | 50.718 |  |  |  |
| 4 | 1.656 | 7.525 | 58.243 |  |  |  |
| 5 | 1.280 | 5.817 | 64.060 |  |  |  |
| Extraction Method: Maximum likelihood method. | | | | | | |
