# Supplementary material for: Developing and validating a questionnaire to assess an individual’s perceived risk of four major non-communicable diseases in Myanmar
Source: PLoS One. 2021 Apr 27;16(4):e0234281. doi: 10.1371/journal.pone.0234281 (PMC8078785; doi:10.1371/journal.pone.0234281)
Supplement: S8 Table — (DOCX) [file pone.0234281.s008.docx]

**S8 Table. Validated final 21-item questionnaire (NCD-PR5-21)**

**English version**

| 1. Strongly disagree 2. Disagree 3. Agree 4. Strongly agree | | | | | | | | |
| --- | --- | --- | --- | --- | --- | --- | --- | --- |
| **Subscale** | **Item** | | | | **Answer** | | | |
| Perceived susceptibility | **Sus_5**. I would likely suffer from any of the NCDs. | | | | 1 | 2 | 3 | 4 |
|  | **Sus_6**. It is almost sure that I would suffer from NCDs. | | | | 1 | 2 | 3 | 4 |
|  | **Sus_8**. There is a possibility that I would suffer from NCDs at this moment. | | | | 1 | 2 | 3 | 4 |
|  | **Sus_10**. I feel I will suffer from an NCD sometime during my life. | | | | 1 | 2 | 3 | 4 |
| Perceived benefit | **Bene_2.** Doing physical exercises can prevent NCDs. | | | | 1 | 2 | 3 | 4 |
|  | **Bene_3.** Eating a healthy diet can prevent NCDs. | | | | 1 | 2 | 3 | 4 |
|  | **Bene_4.** The reduction of drinking an excessive amount of alcohol can prevent NCDs. | | | | 1 | 2 | 3 | 4 |
|  | **Bene_5.** The regular medical checkup is necessary to get an early diagnosis of NCD. | | | | 1 | 2 | 3 | 4 |
|  | **Bene_7.** Living stress-free can prevent NCDs. | | | | 1 | 2 | 3 | 4 |
| Perceived barrier | **Bar_4.** I don’t know about the suitable physical exercises which would help reduce the possibility of suffering from NCDs. | | | | 1 | 2 | 3 | 4 |
|  | **Bar_5.** On most of the days of a week, I don’t have time to do a physical activity of 30 minutes a day. | | | | 1 | 2 | 3 | 4 |
|  | **Bar_7.** I don’t know which type of diet can prevent NCDs. | | | | 1 | 2 | 3 | 4 |
| 1. Not at all confident 2. Somewhat confident 3. Moderately confident 4.Completely confident | | | | | | | | |
| Perceived self-efficacy | **Effi_2.** How much extent do you believe in yourself to live healthily to prevent NCDs? | | | | 1 | 2 | 3 | 4 |
|  | **Effi_3.** How much extent do you believe in yourself to maintain suitable body weight by doing regular physical activity to prevent NCDs? | | | | 1 | 2 | 3 | 4 |
|  | **Effi_7.** How much extent do you believe in yourself to eat a healthy diet only? | | | | 1 | 2 | 3 | 4 |
|  | **Effi_8.** How much extent do you believe in yourself to reduce the risk of NCDs? | | | | 1 | 2 | 3 | 4 |
|  | **Effi_9.** How much extent do you believe in yourself to practice healthy habits? | | | | 1 | 2 | 3 | 4 |
| 1. Strongly disagree | | 1. Disagree | 1. Agree | 1. Strongly agree | | | | |
| Intention to change behavior or cues to action | **Intent_1.** Supposed I am addicted to smoking/betel chewing; I will quit smoking/betel chewing to prevent NCDs. | | | | 1 | 2 | 3 | 4 |
|  | **Intent_2.** I will maintain suitable bodyweight to prevent NCDs. | | | | 1 | 2 | 3 | 4 |
|  | **Intent_3.** I will do physical activities actively to prevent NCDs. | | | | 1 | 2 | 3 | 4 |
|  | **Intent_4.** I will reduce (or) quit alcohol drinking to prevent NCDs. | | | | 1 | 2 | 3 | 4 |

**“မကူးစက်နိုင်သောရောဂါများ ဖြစ်ပွားနိုင်ချေရှိမှုအပေါ် လူပုဂ္ဂိုလ်တစ်ဉီး၏ ခံယူချက်အား ဆန်းစစ်သည့် မေးခွန်းလွှာ”**

| ၁= လုံးဝသဘောမတူပါ။ ၂= သဘောမတူပါ။ ၃= သဘောတူပါသည်။ ၄= လုံးဝသဘောတူပါသည်။ | | | | | |
| --- | --- | --- | --- | --- | --- |
| Perceived susceptibility | Sus_5. ကျွန်ုပ်တွင် မကူးစက်နိုင်သောရောဂါ တစ်မျိုးမျိုး ဖြစ်ပွားနိုင်ချေရှိသည်။ | ၁ | ၂ | ၃ | ၄ |
|  | Sus_6. ကျွန်ုပ်တွင် မကူးစက်နိုင်သောရောဂါ ဖြစ်ပွားလာမည်ဟု သေချာသလောက် ရှိသည်။ | ၁ | ၂ | ၃ | ၄ |
|  | Sus_8. ယခုလက်ရှိ အချိန်၌ပင် ကျွန်ုပ်တွင် မကူးစက်နိုင်သောရောဂါ ဖြစ်ပွားနိုင်ချေ  ရှိသည်။ | ၁ | ၂ | ၃ | ၄ |
|  | Sus_10. ကျွန်ုပ်ဘဝ၏တစ်ချိန်ချိန်တွင် မကူးစက်နိုင်သောရောဂါ ဖြစ်လာ လိမ့်မည်။ | ၁ | ၂ | ၃ | ၄ |
| Perceived benefit | Bene_2. ကိုယ်လက်လှုပ်ရှား လေ့ကျင့်ခန်းလုပ်ခြင်းဖြင့် မကူးစက်နိုင်သော ရောဂါဖြစ်ပွားမှုမှ ကာကွယ်နိုင်စွမ်းရှိသည်။ | ၁ | ၂ | ၃ | ၄ |
|  | Bene_3. ကျန်းမာရေးနှင့်ညီညွတ်သော အစားအစာများကို စားသုံးခြင်းဖြင့် မကူးစက်နိုင် သော ရောဂါဖြစ်ပွားမှုမှ ကာကွယ်နိုင်စွမ်းရှိသည်။ | ၁ | ၂ | ၃ | ၄ |
|  | Bene_4. အရက် အလွန်အကျွံ​သောက်သုံးခြင်းအား လျှော့ချခြင်းသည် မကူးစက်နိုင်သော ရောဂါ ဖြစ်ပွားနိုင်​ချေကို နည်းပါး​စေနိုင်သည်။ | ၁ | ၂ | ၃ | ၄ |
|  | Bene_5. ကျန်းမာရေး ပုံမှန်စစ်ဆေးခြင်းဖြင့် မကူးစက်နိုင်သောရောဂါကို စောစောစီးစီး ရှာဖွေတွေ့ရှိ နိုင်သည်။ | ၁ | ၂ | ၃ | ၄ |
|  | Bene_7. စိတ်သောကကင်းဝေးအောင် နေထိုင်ခြင်းဖြင့် မကူးစက်နိုင်သောရောဂါများ ဖြစ်ပွားမှုမှ ကာကွယ်နိုင်စွမ်းရှိသည်။ | ၁ | ၂ | ၃ | ၄ |
| Perceived barrier | Bar_4. မကူးစက်နိုင်သောရောဂါ ဖြစ်လာနိုင်သော အန္တရာယ်ကိုလျှော့ချရန် အထောက် အကူဖြစ်စေမည့်သင့်လျော်သော ကိုယ်လက်လှုပ်ရှားလေ့ကျင့်ခန်းများကို မသိပါ။ | ၁ | ၂ | ၃ | ၄ |
|  | Bar_5. ရက်သတ္တပတ်တစ်ပတ်၏နေ့ရက် အများစုတွင် ကျွန်ုပ်သည် တစ်ရက်လျှင် မိနစ် ၃၀ ကိုယ်လက်လှုပ်ရှားမှု ပြုလုပ်ရန် အချိန် မရှိပါ။ | ၁ | ၂ | ၃ | ၄ |
|  | Bar_7. ကျွန်ုပ်သည် မကူးစက်နိုင်သောရောဂါကို ကြိုတင် ကာကွယ်နိုင်သည့် စားသောက်မှု ပုံစံအား မသိပါ။ | ၁ | ၂ | ၃ | ၄ |
| ၁= လုံးဝမယုံကြည်ပါ။ ၂= အနည်းငယ်ယုံကြည်ပါသည်။ ၃= အတော်အသင့်ယုံကြည်ပါသည်။ ၄= လုံးဝယုံကြည်ပါသည်။ | | | | | |
| Perceived self-efficacy | Effi_2. မကူးစက်နိုင်သောရောဂါများကို ကာကွယ်ရန် ကျန်းမာသော လူနေမှုပုံစံနှင့်အညီ ပြုမှုနေထိုင်နိုင်လိမ့်မည်ဟု သင်မည်မျှ ယုံကြည်မှုရှိသနည်း။ | ၁ | ၂ | ၃ | ၄ |
|  | Effi_3. မကူးစက်နိုင်သောရောဂါများကို ကာကွယ်နိုင်ရန် ကိုယ်လက်လှုပ်ရှားမှုပုံမှန် လုပ်ခြင်းဖြင့် သင့်​တော်​သော ကိုယ်အလေးချိန်ကို ထိန်းသိမ်း ထားနိုင်မည်ဟု သင်မည်မျှ ယုံကြည်မှုရှိသနည်း။ | ၁ | ၂ | ၃ | ၄ |
|  | Effi_7. ကျန်းမာရေးနှင့်ညီညွတ်မျှတသော အစားအစာများကိုသာ စာသုံး​တော့မည်ဟု သင်မည်မျှ ယုံကြည်မှုရှိသနည်း။ | ၁ | ၂ | ၃ | ၄ |
|  | Effi_8. မကူးစက်နိုင်သောရောဂါ ဖြစ်ပွားခြင်း အန္တရာယ်ကို ထိန်းချုပ်လျှော့ချနိုင်မည်ဟု သင်မည်မျှ ယုံကြည်မှုရှိသနည်း။ | ၁ | ၂ | ၃ | ၄ |
|  | Effi_9. ကျန်းမာ​ရေးနှင့်ညီညွတ်သော အလေ့အထများကို ကျင့်သုံးနိုင်မည်ဟု သင်မည်မျှ ယုံကြည်မှုရှိသနည်း။ | ၁ | ၂ | ၃ | ၄ |
| ၁= လုံးဝသဘောမတူပါ ၂= သဘောမတူပါ ၃= သဘောတူပါသည် ၄= လုံးဝသဘောတူပါသည် | | | | | |
| Behavior change intention | Intent_1. အကယ်၍ ဆေးလိပ်/ကွမ်းစွဲ​နေသူတစ်ဉီး ဖြစ်ခဲ့လျှင် မကူးစက်နိုင်သော ရောဂါများ ကို ကာကွယ်ရန် ဆေးလိပ်/ကွမ်းဖြတ်​တော့မည်။ | ၁ | ၂ | ၃ | ၄ |
|  | Intent_2. ကျွန်ုပ်သည် မကူးစက်နိုင်သောရောဂါများကို ကာကွယ်နိုင်ရန် သင့်​တော်​သော ကိုယ်အလေးချိန်ကို ထိန်းသိမ်း​တော့မည်။ | ၁ | ၂ | ၃ | ၄ |
|  | Intent_3. မကူးစက်နိုင်သောရောဂါများကို ကာကွယ်နိုင်ရန် ကျွန်ုပ်သည် ကိုယ်လက် လှုပ်ရှား မှုကိုတက်ကြွစွာ ဆောင်ရွက်တော့မည်။ | ၁ | ၂ | ၃ | ၄ |
|  | Intent_4. ကျွန်ုပ်သည် မကူးစက်နိုင်သောရောဂါများကို ကာကွယ်နိုင်ရန် အရက် အလွန်အကျွံ သောက်ခြင်းအား ​လျော့ချတော့မည်(သို့) အရက်အားဖြတ်တော့မည်။ | ၁ | ၂ | ၃ | ၄ |
