# Supplementary material for: Developing and validating a questionnaire to assess an individual’s perceived risk of four major non-communicable diseases in Myanmar
Source: PLoS One. 2021 Apr 27;16(4):e0234281. doi: 10.1371/journal.pone.0234281 (PMC8078785; doi:10.1371/journal.pone.0234281)
Supplement: S9 Table — (DOCX) [file pone.0234281.s009.docx]

**S9 Table. Factor loading results and internal reliability of the factors of the final EFA model using all 360 participants**

| **Item** | **Factor** | | | | |
| --- | --- | --- | --- | --- | --- |
|  | **PerIntent** | **PerEffi** | **PerBene** | **PerSus** | **PerBar** |
| intent_1 | **.778** | -.140 | -.010 | -.070 | .019 |
| intent_2 | **.757** | .043 | .034 | -.029 | .037 |
| intent_4 | **.755** | -.139 | .064 | -.001 | -.045 |
| intent_3 | **.717** | .074 | .026 | .024 | .055 |
| intent_5 | **.616** | .165 | -.032 | -.005 | .001 |
| intent_6 | **.551** | .198 | -.049 | .076 | -.021 |
| effi_8 | -.063 | **.755** | .103 | -.071 | .075 |
| effi_9 | .045 | **.744** | -.008 | -.045 | .009 |
| effi_7 | .008 | **.742** | -.011 | -.010 | -.014 |
| effi_2 | -.007 | **.681** | -.036 | .056 | -.077 |
| effi_3 | .026 | **.548** | -.013 | .106 | -.098 |
| bene_2 | -.081 | .069 | **.801** | -.007 | .089 |
| bene_3 | -.013 | .092 | **.735** | -.080 | .084 |
| bene_5 | .039 | -.036 | **.702** | .054 | -.063 |
| bene_4 | .047 | -.001 | **.701** | -.020 | .012 |
| bene_6 | .086 | -.108 | **.603** | .085 | -.171 |
| sus_6 | .005 | .033 | -.065 | **.741** | .011 |
| sus_8 | -.036 | -.007 | -.009 | **.685** | .046 |
| sus_5 | -.080 | .022 | -.015 | **.683** | .039 |
| sus_10 | .081 | -.043 | .101 | **.646** | -.046 |
| sus_3 | -.002 | .007 | .021 | **.556** | .039 |
| bar_4 | .002 | .011 | -.071 | -.013 | **.749** |
| bar_7 | .091 | -.087 | .087 | .050 | **.602** |
| bar_5 | -.040 | -.021 | -.013 | .067 | **.545** |
| **Cronbach's α** | 0.853 | 0.831 | 0.841 | 0.795 | 0.665 |
| **% of variance** | 22.81 | 9.214 | 9.383 | 4.068 | 4.266 |
| **Cumulative %** | 22.81 | 32.025 | 41.408 | 45.475 | 49.741 |

Items were removed if the loadings were below 0.5. Extraction method – Maximum likelihood, Rotation method – Promax with Kaiser Normalization
